# Enhanced surface area and reduced pore collapse of methylated, imine-linked covalent organic frameworks†

**DOI:** 10.1039/d1nr05911d

**Published:** 2021-11-09

**Authors:** Ellen Dautzenberg, Milena Lam, Guanna Li, Louis C. P. M. de Smet

**Affiliations:** Laboratory of Organic Chemistry, Wageningen University Stippeneng 4 6708WE Wageningen The Netherlands louis.desmet@wur.nl; Biobased Chemistry and Technology, Wageningen University Bornse Weilanden 9 6708WG Wageningen The Netherlands

## Abstract

Covalent Organic Frameworks (COFs) are thermally and chemically stable, nanoporous materials with high surface areas, making them interesting for a large variety of applications including energy storage, gas separation, catalysis and chemical sensing. However, pore blocking and pore collapse may limit their performance. Reducing the capillary forces by using solvents with low surface tension, like supercritical CO_2_, for activation, and the introduction of bulky isopropyl/methoxy groups were found to reduce pore collapse. Herein, we present an easy-to-use alternative that involves the combination of a new, methylated building block (2,4,6-trimethylbenzene-1,3,5-tricarbaldehyde, Me_3_TFB) with vacuum drying. Condensation of Me_3_TFB with 1,4-phenylenediamine (PA) or benzidine (BD) resulted in imine-linked 2D COFs (Me_3_TFB-PA and Me_3_TFB-BD) with higher degrees of crystallinity and higher BET surface areas compared to their non-methylated counterparts (TFB-PA and TFB-BD). This was rationalized by density functional theory computations. Additionally, the methylated COFs are less prone to pore collapse when subjected to vacuum drying and their BET surface area was found to remain stable for at least four weeks. Within the context of their applicability as sensors, we also studied the influence of hydrochloric acid vapour on the optical and structural properties of all COFs. Upon acid exposure their colour and absorbance spectra changed, making them indeed suitable for acid detection. Infrared spectroscopy revealed that the colour change is likely attributed to the cleavage of imine bonds, which are only partially restored after ammonia exposure. While this limits their application as reusable sensors, our work presents a facile method to increase the robustness of commonly known COFs.

## Abstract 2

Methylation of aldehyde nodes in Covalent Organic Frameworks leads to enhanced BET surface areas and reduced pore collapse compared to their non-methylated counterparts, which has been rationalized by DFT computations.

## Graphical abstract

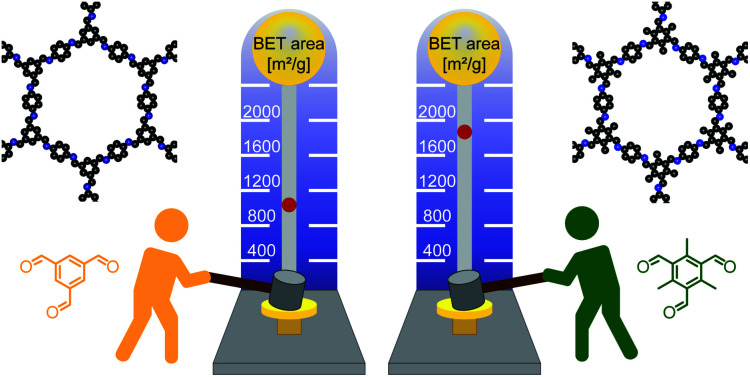

## Introduction

Covalent Organic Frameworks (COFs) are a class of nanoporous materials first discovered in 2005 by Yaghi and co-workers.^1^ Since then, COFs have gained increasingly more interest and a huge variety of different materials and applications have been reported.^2,3^ COFs consist of fully organic building blocks linked by dynamic covalent chemistry.^4,5^ Dynamic covalent chemistry refers to reversible reactions carried out under thermodynamic reaction conditions. This enables error correction, leading to crystalline frameworks with a long-range order. The symmetry of the building blocks is determining the crystal structure. In the case of 2D COFs, the third dimension is formed by π–π stacking. The layers usually stack in an eclipsed structure leading to channels in the framework, which results in high surface areas. These high surface areas are of interest for, amongst others, energy storage, gas separation, catalysis, and chemical sensing.^3,6–8^ The variability in organic building blocks in terms of functional groups or symmetry allow a high control over the COF properties and thus leads to tailor-made materials. However, the boronic esters used to make the first COFs were susceptible to hydrolysis. This has been improved by using imine-linked COFs or even more stable β-ketoenamine COFs. The latter first forms imine bonds, which then tautomerise irreversibly into the β-ketoenamine linkage. The irreversible tautomerisation results in a lower crystallinity and surface area compared to their imine counterparts.^2^ The chemical stability of COFs does not only depend on the type of linking chemistry, but can also be affected by the nature of the core of the organic building blocks. Ma *et al.*^9^ reported two highly porous 3D COFs of terephthalaldehyde or 4,4′-biphenyldicarboxaldehyde and a tetrahedral amine with isopropyl groups in *ortho* position. Isopropyl groups are hydrophobic and their steric demand protects the imine groups from hydrolysis. The COFs are reported to be stable in acidic and strong alkaline solutions without a decrease in surface area and crystallinity. Such stability was also achieved by methoxy substitution of the aldehyde node.^10^ In another study, Wang *et al.*^11^ reported enhanced BET surface areas for *ortho* methoxy-substituted aldehyde nodes and *meta* methyl-substituted amine linkers.

COF stability is not only about the chemical stability of the functional groups involved, but also about the stability of the physical properties such as pore size, pore shape and surface area. It has been identified that the conditions for work-up and COF activation are crucial to get accessible pores and high surface areas. After synthesis, COFs are most commonly washed with several solvents to remove impurities and water from the pores prior to drying. Vacuum drying has often been used, but was identified to induce partial pore collapse due to capillary forces.^12^ Capillary forces increase with decreasing pore size and COFs with typical pore sizes around a few nanometres are therefore subjected to strong capillary forces. To overcome pore collapse, milder activation methods such as supercritical CO_2_ drying^12^ or washing with ultra-low surface tension solvents prior to drying^13^ were suggested to keep the framework intact upon activation. Feriante *et al.*^12^ and Zhu *et al.*^13^ both reported methoxy-functionalised, imine-based COFs, which were less prone to pore collapse compared to the non-methoxylated equivalent.

In the current paper, we introduce methyl groups in the synthesis of imine-based COFs as alternative to the bulky isopropyl and the smaller methoxy groups. Depending on the COF application, the use of this smaller – but still hydrophobic – group can be advantageous as it has less impact on the pore size.

Herein, we present 2,4,6-trimethylbenzene-1,3,5-tricarb-aldehyde (Me_3_TFB) as building block for two novel COFs, namely Me_3_TFB-PA and Me_3_TFB-BD, which are compared with two reference COFs TFB-PA^6^ and TFB-BD.^14,15^ The methylation significantly increases the BET surface area by a factor of 1.8 and 1.4, respectively. The physical stability of the framework towards pore collapse during vacuum activation also profits from the methylation. This work shows a facile approach in stabilizing COFs by methylating the aldehyde. The novel COFs maintain their high surface area on the bench for at least four weeks. Finally, the effect of acid vapour on the optical and structural properties of the COFs was studied.

## Results and discussion

### Synthetic procedures

**Scheme 1:**
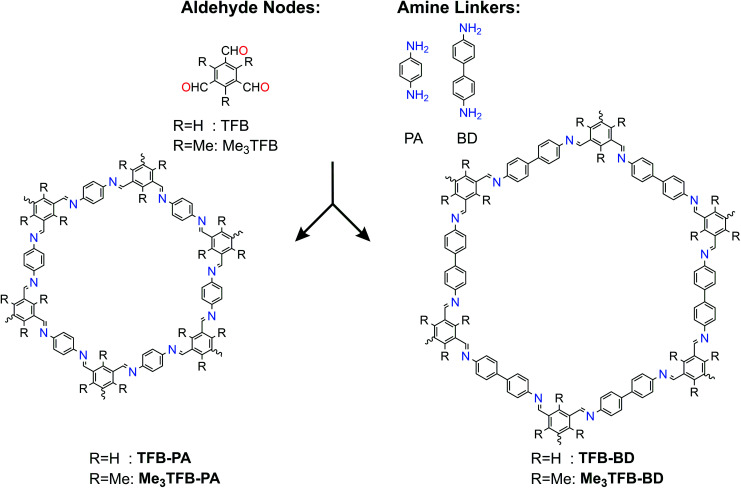
Different aldehyde and amine building blocks used for introducing methyl groups in COFs and their resulting structures.

1,3,5-Benzenetricarbaldehyde (TFB) and 2,4,6-trimethyl-benzene-1,3,5-tricarbaldehyde (Me_3_TFB) were condensed with two different amines, namely 1,4-phenylenediamine (PA) and benzidine (BD) (Scheme 1) to yield the novel 2D imine-linked COFs (Me_3_TFB-PA and Me_3_TFB-BD) and their non-methylated equivalents (TFB-PA and TFB-BD). Each COF has been synthesised and characterised thrice. We selected this set of COFs, because TFB-PA and TFB-BD have been widely studied in literature.^6,14–16^ The synthesis was carried out at 70 °C and atmospheric pressure with acetic acid as catalyst and water to enhance dynamic covalent chemistry in a mixture of mesitylene : 1,4-dioxane 1 : 4 v/v for three days.^17^ A washing protocol from Vitaku *et al.* has been used for the work-up.^18^ Afterwards, half of the obtained powder was dried overnight in a vacuum-oven at 120 °C and the other half at 120 °C in a regular, air-ventilated oven before the COFs were characterised. The characterisation for the reference COFs can be found in the ESI (Fig. S1, S2, S5, S6, S9 and S10†).

### Characterisation

**Fig. 1:**
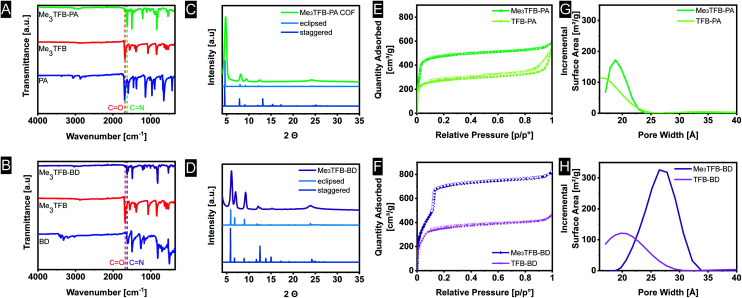
Characteristics of Me_3_TFB-PA (top) and Me_3_TFB-BD (bottom): FT-IR spectra of COFs and starting materials (A and B), PXRD spectra including simulated PXRD patterns (C and D), nitrogen sorption isotherms including their non-methylated references (E and F) and pore size distributions (G and H).

FT-IR spectroscopy on the novel COFs confirmed the formation of imine bonds (Fig. 1A). Me_3_TFB-PA shows a characteristic imine stretch at 1623 cm^−1^, demonstrating a successful reaction. A weak band at 1693 cm^−1^ indicates low fractions of aldehyde bonds. This IR characteristic has also been reported by others and may origin from unreacted side-groups at the outside of the 2D polymeric sheet.^12,13,19^

**Fig. 2:**
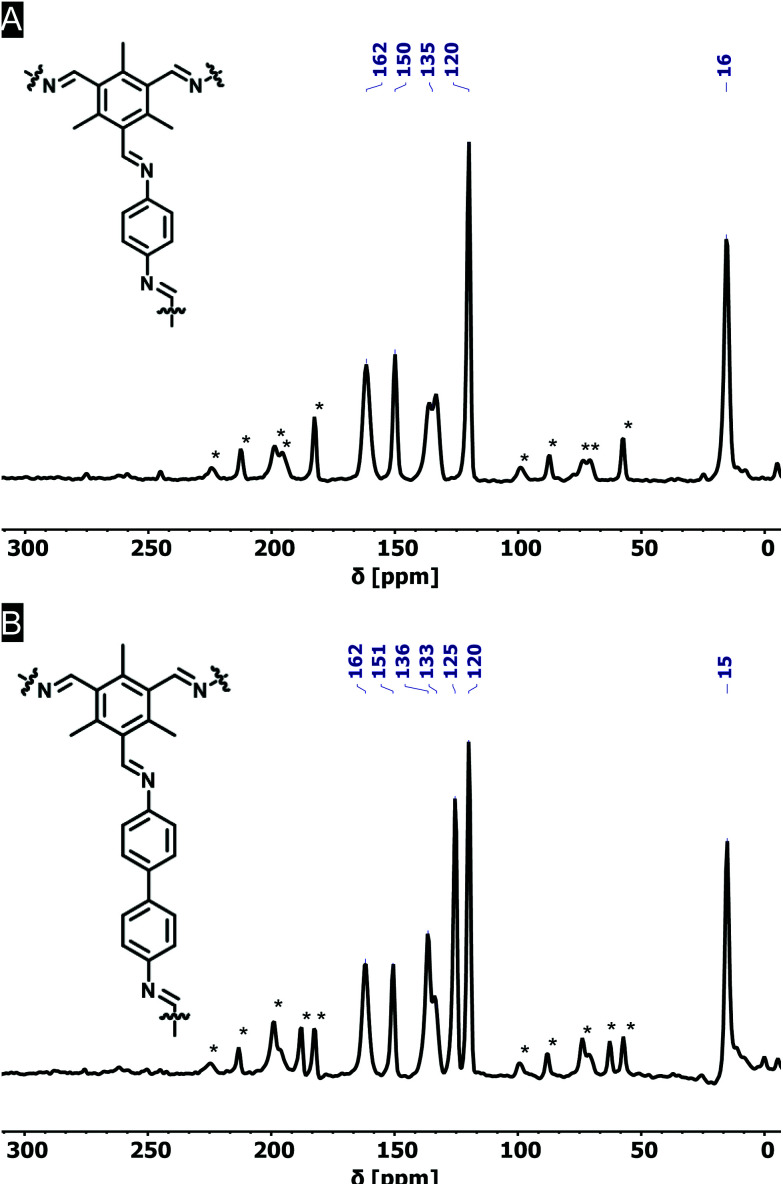
Solid-state NMR spectra of Me_3_TFB-PA (A) and Me_3_TFB-BD (B). The signal at 162 ppm can be assigned to the carbon of an imine-bound nitrogen, indicating the formation of imine bonds. Spinning side bands, here indicated with an asterisk, were determined by comparing different MAS frequencies.

The formation of imine bonds was also confirmed by ^13^C cross-polarization magic angle spinning solid-state NMR (^13^C CPMAS ssNMR) (Fig. 2A) as the signal at 162 ppm is typical for imine carbons. The signal at 194 ppm points to aldehyde carbons, but compared to the starting compound this is strongly attenuated and just the above noise level.

Comparable FT-IR results can be found for Me_3_TFB-BD COF with an imine band at 1627 cm^−1^ and a small carbonyl stretch at 1693 cm^−1^ (Fig. 1B). Again, an ssNMR signal at 162 ppm confirms the formation of an imine (Fig. 2B). All FT-IR spectra of the triplicates can be found in the ESI (Fig. S3 and S4†).

Thermogravimetric analysis revealed that Me_3_TFB-PA is thermally stable up to 355 °C and Me_3_TFB-BD up to 390 °C (ESI Fig. S15†), which is in line with several previously reported imine-linked COFs.^6,14,20,21^

Powder X-Ray Diffraction (PXRD) was performed on all samples to determine the crystallinity of the materials. The obtained spectra were compared to the simulated diffraction patterns of optimized crystal structures obtained from density-functional theory (DFT) calculations (coordinates in ESI†). Me_3_TFB-PA COF displays several diffraction peaks, which are clearly baseline separated from each other at 4.8°, 8.2°, 9.4°, 12.5° and a broader signal around 24° (Fig. 1C). Two different stacking structures have been computed for comparison: an eclipsed and a staggered structure. Comparing peak positions and relative intensities of the peaks, it can be concluded that Me_3_TFB-PA COF crystallises in an eclipsed stacking conformation. For all three samples, Scherrer analysis was performed and gave an estimated average domain size of 15.1 ± 1.5 nm. The PXRD spectrum of Me_3_TFB-BD COF also shows several narrow diffraction peaks, especially at low angles: 3.6°, 6.1° 7.0°, 9.3°, 12.1°, 12.6°, 15.3° (Fig. 1D). The peak at 3.6° was confirmed by a second low-angle PXRD measurement with different settings, which only scanned angles from 1.5–10° to be able to detect signals in this low-angle region without detecting the source. The spectrum can be found in the ESI (Fig. S8†) as well as the low-angle spectrum of Me_3_TFB-PA (ESI Fig. S7†). Scherrer analysis for this sample results in an approximate domain size of 20.5 ± 2.1 nm. Comparison with the computed PXRD diffraction patterns leads again to a preferred eclipsed stacking.

### BET surface area

Nitrogen sorption measurements were carried out to determine the surface area of the COFs (ESI Fig. S11–S14†). For Me_3_TFB-PA, the adsorption–desorption isotherm can be classified as a type I isotherm (Fig. 1E).^22^ This is characteristic for microporous materials of which most of the surface area is internal surface area within the framework. The micropores lead to rapid pore filling at low pressures and a nearly horizontal plateau with increasing relative pressures. Also TFB-PA yields a type I isotherm, including an H4 hysteresis loop, which is absent for Me_3_TFB-PA. Hysteresis loops can be a result of capillary condensation and/or pore blocking.^23^ The pore volume was determined in the adsorption branch at *p*/*p*^0^ = 0.95, yielding a value of 0.88 ± 0.04 cm^3^ g^−1^. The BET surface area (*S*_BET_) was calculated using the Rouquerol criteria^24^ to apply the BET theory to microporous materials. For all samples of the same COF, the same pressure range was used for the linear regression. Afterwards, the obtained BET surface areas were averaged. For Me_3_TFB-PA *S*_BET_ was found to be 1877 ± 20 m^2^ g^−1^. To determine the pore size distribution, HS-2D-NLDFT analysis was carried out using the isotherm data and a model with cylindrical micropores for carbon surfaces with N_2_. Me_3_TFB-PA COF shows a narrow pore size distribution with a clear maximum at 1.9 nm (Fig. 1G). The pore size is slightly larger compared to TFB-PA, which exhibits a broader pore size distribution with only one maximum at 1.7 nm.

Me_3_TFB-BD COF on the other hand, has a type IVb isotherm (Fig. 1F). The microporosity is shown by a large adsorption of nitrogen in the low pressure range (*p*/*p*^0^ < 0.1). The step in the isotherm at *p*/*p*^0^ = 0.1 indicates capillary condensation. The lack of hysteresis is in line with the cylindrical shape of the pores.^22^ The pore volume at *p*/*p*^0^ = 0.95 and BET surface areas were measured to be 1.24 ± 0.03 cm^3^ g^−1^ and 2115 ± 50 m^2^ g^−1^, respectively, while the pore size distribution has a narrow maximum at 2.7 nm (Fig. 1H). With this analysis, where the Rouquerol criteria^24^ were prioritised, *R*^2^ values in the range of 0.996–0.997 were found for the benzidine COFs. The same HS-2D-NLDFT model has been used for the pore size distribution calculation again. Comparing the pore size distribution of Me_3_TFB-BD with the pore size distribution of the non-methylated TFB, which shows a pore size of 2.1 nm, results again in a larger pore size for the methylated COF.

**Fig. 3:**
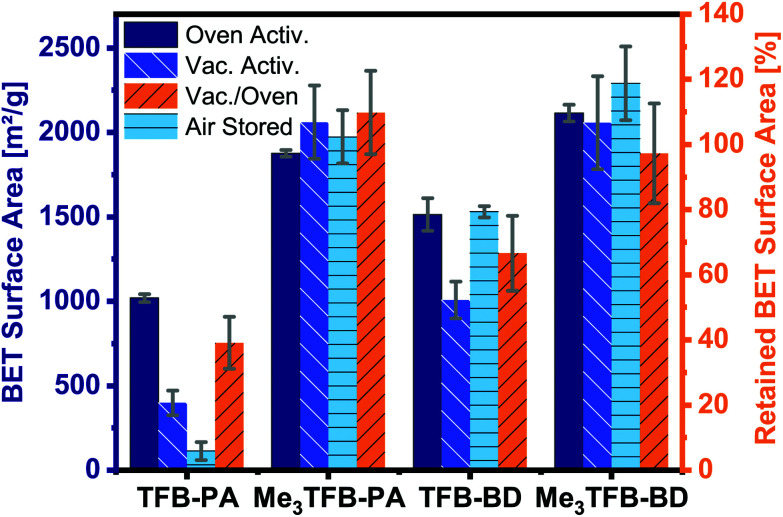
BET surface areas with different activation methods (oven-dried and vacuum-dried) and after four weeks stored on the bench. The retained BET surface area gives the ratio how much *S*_BET_ has been left after vacuum drying. The errors result from the analyses of three different batches.

By comparing the BET surface area of the methylated COFs with their non-methylated equivalents after oven activation, it can be clearly seen that the methylated aldehyde building block significantly increases the BET surface area (Fig. 3). The methylated building block increases the BET surface area by a factor of 1.8 and 1.4 for TFB-PA and TFB-BD, respectively. We would have expected that two *ortho* methyl groups would sterically hinder the reaction, which would have resulted in lower BET surface areas. Therefore, we used theoretical modelling to further study this increase in BET surface area. The crystal structures of DFT optimization reveal the *C*_3_ symmetric incorporation of the aldehyde building block into the framework (ESI Fig. S16 and S17†). Additionally, TFB and Me_3_TFB were geometry optimized by DFT calculations and the rotational barriers for the rotation of the carbonyl groups were modelled (ESI Fig. S18 and S19†). It was found that the methyl groups hinder the full conjugation of the carbonyl groups with the π-system of the aromatic Me_3_TFB ring, leading to a non-planar structure with lower symmetry compared to TFB. For methylated and non-methylated aldehydes, the resulting rotational energy profiles show two minima with a difference in energy of 4.2 kJ mol^−1^, which means both conformations can be present in the system at 70 °C during the reaction. The rotational barrier, which has to be overcome to change conformation, is lower for Me_3_TFB compared to TFB. This is due to the enhanced conjugation and full symmetry in TFB while Me_3_TFB does not have such a strong conjugation and symmetry. The rotational barrier is hence smaller and the reaction rate for the conformational change is magnitudes higher. This implies that, during COF condensation, favoured *C*_3_ symmetric aldehyde conformations are re-formed faster from the *C*_S_ symmetric conformation for Me_3_TFB, which is in line with the experimentally found larger domain sizes and higher BET surface areas. Furthermore, to evaluate the effect of methyl groups on the stability of the thus-formed Me_3_TFB-BD and Me_3_TFB-PA COF materials, the formation energies all for COFs were calculated according to the reaction formula shown in ESI Fig. S20†, based on the assumption that all aldehyde groups react with all amine groups to form the imine-linked COF. The results (Table S5†) indicate that formation of both Me_3_TFB-BD and Me_3_TFB-PA, *i.e.* the COFs featured with methyl groups on the aldehyde node, are thermodynamically more favourable than their non-methylated counterparts, indicating a higher stability of the methylated COFs compared to the non-methylated COFs.

To study whether the addition of methyl groups also results in a more stable framework, we studied the pore collapse by re-measuring the BET surface area of all COFs after vacuum activation (120 °C, vacuum-oven, overnight). To correct for the difference in BET surface area due to different pore sizes, the retained BET surface area has been calculated as the ratio of the vacuum-activated *S*_BET_ over the oven-activated *S*_BET_, given in area in percentages. Both methylated COFs have a higher BET surface area compared to their non-methylated equivalents (Me_3_TFB-PA, *S*_BET_ = 2061 ± 218 m^2^ g^−1^; Me_3_TFB-BD, *S*_BET_ = 2057 ± 276 m^2^ g^−1^; TFB-PA, *S*_BET_ = 398 ± 72 m^2^ g^−1^; TFB-BD, *S*_BET_ = 1008 ± 109 m^2^ g^−1^) after vacuum activation (Fig. 3). The retained BET surface area for TFB-PA is only 39 ± 8%, which means the framework collapses when exposed to vacuum. A comparable trend, though less prominent, can be observed for TFB-BD with a retained *S*_BET_ of 67 ± 12%. In the case of the methyl-containing COFs, the vacuum activation is qualified as the better method for Me_3_TFB-PA COF (retained *S*_BET_ 110 ± 13%), while both activation processes are equally good for Me_3_TFB-BD (retained *S*_BET_ 97 ± 15%). In other words, the BET surface areas increase by a factor of 2.8 and 1.4 for Me_3_TFB-PA and Me_3_TFB-BD, respectively. The methylated COFs are hence less prone to pore collapse.

In addition, we noticed that the BET surface area of TFB-PA decreases already drastically to 113 ± 53 m^2^ g^−1^ in the first four weeks, which means only 11% of the original BET surface area are accessible after one month. Therefore, we investigated the air stability of the other three COFs as well, by leaving the oven-activated samples for four weeks in an open vial on the bench, before determining the BET surface area again. These three COFs did not show such a large drop in the *S*_BET_ values. Instead, on average a slight increase is observed (up to 8% for Me_3_TFB-BD), although the differences are within the standard deviation. In any case, as no decrease is observed, we conclude that TFB-BD, Me_3_TFB-PA, and Me_3_TFB-BD are stable for the reported conditions. Again, this shows that the introduction of methyl groups stabilizes the phenylenediamine COF.

### Acid-vapour sensing

**Fig. 4:**
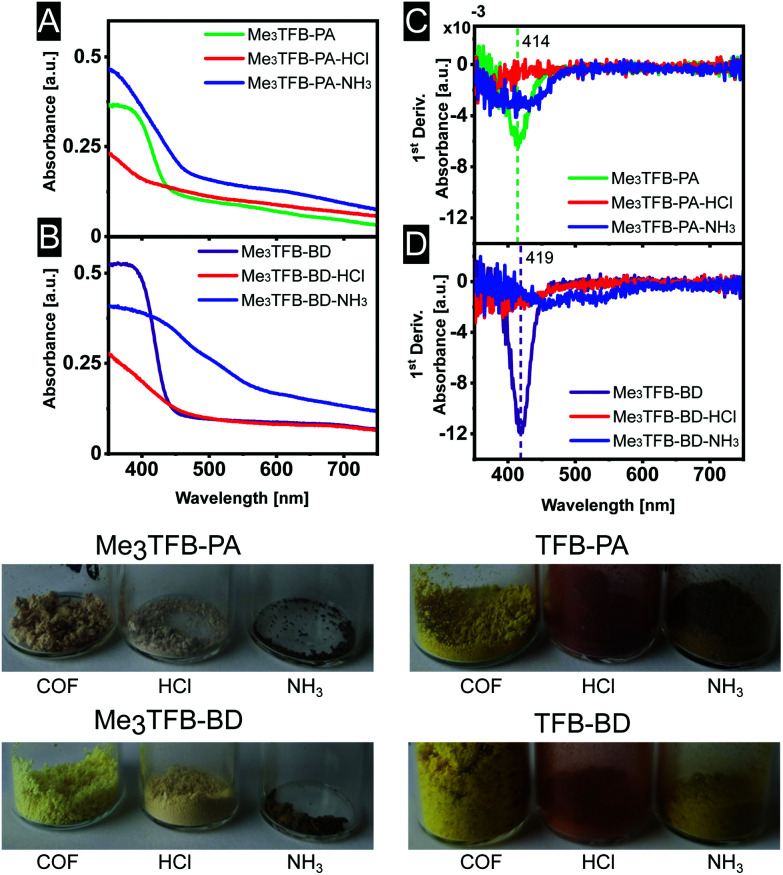
Absorbance spectra (A and B) and their first derivative (C and D) for a better comparison of Me_3_TFB-PA and Me_3_TFB-BD, as well as optical photographs of all stages in the sensing process.

The optical properties of some COFs are reported to respond to changes in acidity and solvent, enabling them to be used as chemical sensors. In more detail, Ascherl *et al.* observed a solvatochromic effect, which has been visible in diffuse reflectance spectroscopy, before employing the pyrene-containing COF for the development of a humidity sensor.^8^ Several studies report on COF-based chemosensors for the detection of gaseous hydrochloric acid,^25,26^ and a series of aqueous Brønsted acids.^26,27^ Recently, Yang *et al.* have studied the effect of imine-protonation on the optical properties of three imine-linked COFs. It was found that all COFs showed a bathochromic shift after acid exposure due to the protonation of the imine bond.^28^ Based on both findings, we looked into the absorption spectra of Me_3_TFB-PA and Me_3_TFB-BD and their response towards hydrochloric acid vapour. To this end, samples of the new COFs were pressed as a thin film between two glass plates. Absorption spectra were collected before treatment with acid by measuring the samples in an integrating sphere and collecting diffuse reflectance and diffuse transmission to calculate the absorbance. Both absorbance spectra have an inverse S-curve shape, start absorbing between 400–450 nm, and have absorption edges around 378 nm for Me_3_TFB-PA and 385 nm for Me_3_TFB-BD (Fig. 4A and B). To facilitate the comparison between the different samples, the inflection point was used and the first derivative was calculated in Origin to determine the minimum (Fig. 4C and D), which were found to be similar for both COFs (414 nm for Me_3_TFB-PA and 419 nm for Me_3_TFB-BD COF).

As the two-plate configuration does not allow vapour exposure experiments, a new sample was taken from the same synthetic COF batch, which was subjected to hydrochloric acid vapour for 30 minutes. Within minutes, a colour change (paler powder for methylated COFs, red for non-methylated equivalents) was observed by the naked eye (Fig. 4), which was confirmed by an absorbance measurement of the acid-exposed COF sample. The original inverted S-curve was blue shifted and additionally became more stretched for methylated COFs. In comparison, the non-methylated COFs were red shifted, but also stretched.

In order to study the applicability of this interesting property, we investigated whether the COF's response towards hydrochloric acid vapour is reversible by subjecting the acid-exposed material towards ammonia vapour for 30 minutes. Within minutes, another colour change could be observed (Fig. 4), back more towards the original yellow, but going even further to brown. This was the case for both methylated and non-methylated COFs. To study this in more detail, the absorbance was once more measured. The absorbance curve resembles more its original shape for Me_3_TFB-PA COF, although the first derivative indicates a wider range in which the absorbance changes and the absorption edge is red shifted with respect to the acid-exposed sample. For Me_3_TFB-BD COF, the spectrum also shifts back to higher wavelength, but even wider. The first derivative indicates two small minima with shifted positions that do not overlap with the original minimum, so the acid has an impact on the COF structure. The absorbance spectra for the non-methylated COFs can be found in the ESI (Fig. S21 and S22†). No change in absorbance could be found for just exposing the samples to ammonia vapour (ESI Fig. S23 and S24†).

**Fig. 5:**
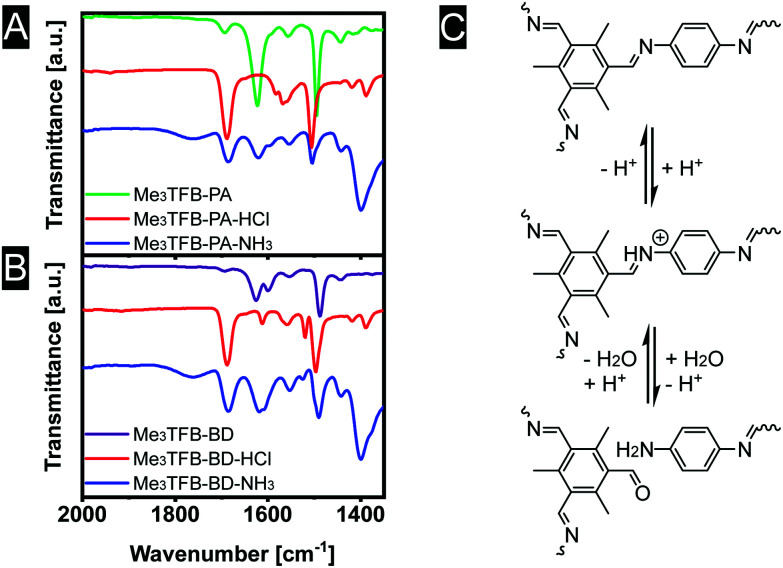
FT-IR spectra (A and B) to elucidate the sensing mechanism (C).

We speculate that the acid protonates the nitrogen atom (Fig. 5C). In a second step, this protonation could lead to a bond cleavage, affecting the COF structure, which is expected to limit the sensor performance. In an attempt to gain insight into the sensing mechanism, FT-IR spectra were recorded in all stages of the sensing process (Fig. 5A and B). Upon exposure to acid vapour the FT-IR spectra do not show any imine stretching vibration anymore. Instead, strongly increased bands at 1688 cm^−1^ are observed, leading to the conclusion that the exposure of acid breaks down the framework, explaining the observed colour changes of the materials. After exposure towards ammonia, equally strong bands near 1621 cm^−1^ (imine stretching vibration) and 1685 cm^−1^ (carbonyl stretching vibration) are visible, indicating a partial recovery of the imine bonds.

## Conclusions

In conclusion, a new methyl-substituted aldehyde building block has been used for COF synthesis, resulting in two novel frameworks, namely Me_3_TFB-PA and Me_3_TFB-BD. They have been compared with non-methylated reference COFs regarding their BET surface area, pore collapse and their applicability as optical acid sensors. With a facile synthetic approach, which does not require any specialized equipment, we found that methylation of the aldehyde node results in enhanced BET surface areas up to *ca.* 2000 m^2^ g^−1^ and high degrees of crystallinity compared to their non-methylated equivalents. The obtained BET surface areas are comparable to the ones achieved by supercritical CO_2_ activation. The higher surface areas and crystallinity are in line with DFT-calculated reaction rates. With respect to TFB, Me_3_TFB loses symmetry and its full conjugation due to the *ortho*-substituted methyl groups. This leads to a smaller rotation barrier towards the favoured *C*_3_ conformation for crystallisation and therefore to higher reaction rates. Additionally, the novel methylated COFs are less prone to pore collapse during vacuum activation and the BET surface area remains stable within at least four weeks.

The COFs can be used as an optical sensor to detect hydrochloric acid based on a change in colour. The sensing mechanism reveals that the colour change is likely attributed to the cleavage of the imine bond, which is partially restored after exposure to ammonia vapour. Even though this limits their applicability as a reusable sensor for hydrochloric acid, our approach enables the facile synthesis of high surface area COFs that are more robust compared to commonly known COFs. The effect of *ortho*-substituted methyl groups of the amine building block and systematically increasing the number of methyl groups on the resulting COF properties are currently under investigation.

## Author contributions

L.C.P.M.d.S. and E.D. conceived the project. E.D. and M.L. designed and performed the experiments. G.L. performed the modelling. E.D., M.L. and L.C.P.M.d.S. analysed all data. L.C.P.M.d.S. guided the project. E.D. wrote the first draft, and all authors gave input.

## Conflicts of interest

There are no conflicts to declare.

## Supplementary Material

NR-013-D1NR05911D-s001

NR-013-D1NR05911D-s002

NR-013-D1NR05911D-s003

NR-013-D1NR05911D-s004

NR-013-D1NR05911D-s005
