# Supplementary material for: Enhanced surface area and reduced pore collapse of methylated, imine-linked covalent organic frameworks
Source: Nanoscale. 2021 Nov 9;13(46):19446–52. doi: 10.1039/d1nr05911d (PMC8638808; doi:10.1039/d1nr05911d)
Supplement: NR-013-D1NR05911D-s001 [file NR-013-D1NR05911D-s001.pdf]

Electronic Supplementary Material (ESI) for Nanoscale.
This journal is © The Royal Society of Chemistry 2021

SUPPORTING INFORMATION

Enhanced Surface Area and Reduced Pore Collapse of Methylated, Imine-Linked Covalent Organic Frameworks

Ellen Dautzenberg[a], Milena Lam[a], Guanna Li[a,b], Louis C. P. M. de Smet[a]*

[a] Laboratory of Organic Chemistry, Wageningen University, Stippeneng 4, 6708WE Wageningen, The Netherlands

[b] Biobased Chemistry and Technology, Wageningen University, Bornse Weilanden 9, 6708WG Wageningen, The Netherlands

Contact details: ellen.dautzenberg@wur.nl (Ellen Dautzenberg), milena.lam@wur.nl (Milena Lam), guanna.li@wur.nl (Guanna Li), louis.desmet@wur.nl (Louis C.P.M. de Smet*, +31-317481268).

Author Contributions: L.C.P.M.d.S. and E.D. devised the project. E.D. and L.M. planned and performed the experiments. G.L. performed the modelling. E.D., M.L. and L.C.P.M.d.S. analyzed the data. L.C.P.M.d.S. guided the project. E.D. wrote the first draft, and all authors gave input.

Contents

1. General Information 3
   Materials 3
   Instrumentation 3
2. Synthetic Procedures 5
   Building blocks: 5
   COF synthesis: 6
3. FT-IR Spectra 11
4. Powder X-Ray Diffraction Analysis 13
5. ^{13}C CPMAS NMR Spectra 15
6. Nitrogen Sorption Analysis 16
7. DFT modelling for conformation analysis and rate constant 20
8. Vapor Experiments 23
9. ^{1}H-NMR and ^{13}C-NMR spectra of building blocks 25
10. Mass Spectrometry 27
11. References 29

1. General Information

Materials

1,4-Phenylenediamine (>98 % (GC)(T)) and 1,3,5-benzenetricarboxaldehyde (96%) were purchased from TCI Europe N.V., benzidine (98%) was purchased form Abcr and all chemicals were used without further purification. Mesitylene (99%, extra pure) was purchased from Fisher Scientific and 1,4-dioxane (99%) was purchased from Acros Organics B.V.B.A. All solvents and glacial acetic acid (AR) were purchased from commercial sources and used without further purifications.

Instrumentation

^{1}H and ^{13}C NMR spectra were recorded on a Bruker AVANCE III NMR spectrometer at 400 MHz and 100 MHz, respectively. The spectra were referenced with respect to the deuterated solvents ($CDCl_3$: 7.26 ppm, 77.16 ppm, DMSO-d_6: 2.5 ppm, 39.52 ppm). ^{1}H and $^{13}C\{^{1}H\}$ cross-polarization magic angle spinning (CPMAS) solid-state NMR (ssNMR) spectra were recorded on a Bruker AVANCE III HD spectrometer at 700.13 MHz (16.4 T) and 176 MHz, respectively. Solid-state NMR samples were packed into 4 mm zirconia rotors and spun at MAS frequencies of 11 kHz and 14 kHz at 298 K. The ^{13}C CPMAS spectra were obtained with a recycle delay of 3 s and a contact time of 3 ms unless stated differently. The ^{13}C ssNMR spectra were referenced with respect to adamantane (^{13}C, 29.456 ppm). The spectra were analyzed using MestReNova (version 14.1.0).

Mass spectrometry data was collected using an Exactive high-resolution MS instrument (Thermo Scientific) equipped with an ESI probe or a DART probe. Pierce™ LTQ ESI Positive/Negative Ion Calibration Solution was used for calibration. Thermo XCalibur software (version) was used for instrument control, data acquisition and data processing. The theoretical mass was calculated with an online calculator (https://www.envipat.eawag.ch/).[1]

FT-IR spectra were obtained on a Bruker Tensor 27 spectrometer with platinum attenuated total reflection accessory. The samples were applied as powder on top of the crystal. 64 scans were performed with a resolution of 4 cm^{-1}.

Powder X-Ray diffraction measurements were recorded with a Philips X'pert-PRO at 40 kV and 40 mA from 4-40° (step size: 0.05°, step time: 90 s, mask in front of entrance: 10 mm and slid 1°, slid before detector: 1°) and from 1.5-10° (step size: 0.05°, step time: 500 s, mask in front of entrance: 5 mm and slid 0.5°, slid before detector: 0.25°). X-Rays were generated by a Cu anode Kα (1.54 λ) radiation. Scherrer analysis was carried out with Diffrac.Eva (version 5.2.0.5) from Bruker with an instrumental width of 0.050 and a Scherrer constant of 0.89. The peak with the lowest angle was used to carry out the analysis.

Nitrogen adsorption-desorption measurements were performed on a MicroActive for Tristar II Plus 2.01 at 77.350 K. Before the measurement, the samples were outgassed at 120 °C overnight. Surface areas were calculated from the adsorption data using Brunauer-Emmet-Teller (BET) methods and Rouquerol criteria. The pore-size distribution curves were obtained from the adsorption branches using the method "HS-2D-NLDFT, Carb Cyl Pores (ZTC) N2@77K". An optimum between goodness of fit and

smoothness of the pore size distribution was aimed for. The average of three different COF batches was used to determine the BET surface areas.

Thermogravimetric analysis was performed on a Perkin Elmer STA 6000. The sample was heated to 30 °C, the temperature was hold for one minute and afterwards the sample was heated with 10.00 °C/min to 700 °C in a nitrogen atmosphere (20 ml/min).

Origin2020b (64-bit) version 9.7.5.184 was used to analyze, to plot and to fit all data.

Computation Details

All DFT calculations for COF structures were performed by using the Vienna Ab initio Simulation Package (VASP, version 5.4.4).[2,3] The PBE functional based on the generalized gradient approximation was chosen to account for the exchange–correlation energy.[4] A plane-wave basis set in combination with the projected augmented wave (PAW) method was used to describe the valence electrons and the valence-core interactions, respectively.[5] The kinetic energy cut-off of the plane wave basis set was set to 500 eV. Gaussian smearing of the population of partial occupancies with a width of 0.05 eV was used during iterative diagonalization of the Kohn-Sham Hamiltonian. The threshold for energy convergence for each iteration was set to 10^{-5} eV. Geometries were assumed to be converged when forces on each atom were less than 0.05 eV/Å. The Brillouin zone integration and k-point sampling were done with a Gamma centered 1*1*8 and 2*2*4 grid points for the eclipsed and staggered unit cells, respectively. The Van der Waals (vdW) interactions were included by using Grimme's DFT-D3(BJ) method as implemented in VASP.[6] Simulated XRD patterns were obtained by using VESTA (version 3.4.8).[7] Coordinates of all crystal structures are provided as separate files. Individual molecules of 1,3,5-triformylbenzene, 2,4,6-trimethylbenzene-1,3,5-tricarbaldehyde, benzidine, 1,4-phenylenediamine and H_2O were optimized in a periodic box of 20Å × 20Å × 20Å without thermochemical correction for the calculation of the formation energy.

DFT calculations for 1,3,5-benzenetricarbaldehyde (TFB) and 2,4,6-trimethylbenzene-1,3,5-tricarbaldehyde (Me_3TFB) were performed by using Gaussian 16.[8] Geometry optimization and dihedral scans were calculated by using B3LYP with a 6-311G(d,p) basis set. For Me_3TFB, symmetry-broken optimization was performed.

2. Synthetic Procedures

Building blocks:

The synthesis of 2,4,6-trimethyl-benzene-1,3,5-tricarbaldehyde is based on a procedure of Van der Made *et al.*[9] and Slater *et al.*[10]:

1,3,5-Tris(bromomethyl)-2,4,6-trimethylbenzene

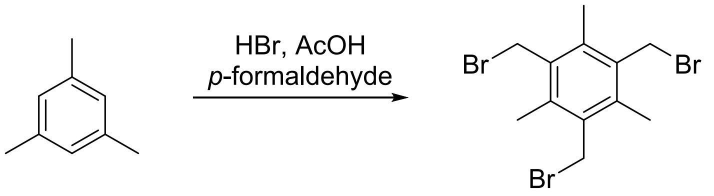

1 **2**

To a vacuum-dried 250 mL round-bottom flask under N_2 atmosphere, mesitylene (**1**) (7.0 mL, 6.9 g, 57 mmol, 1 equiv.) was added, followed by the addition of acetic acid (25 mL, 27 g, 455 mmol, 8 equiv.) and HBr in AcOH (33 wt% solution) (35 mL). Then, *para*-formaldehyde (5.55 g, 185 mmol, 3.2 equiv.) was added and the mixture was stirred at 95 °C overnight. The next morning, a precipitate had formed and the mixture was cooled down to room temperature and crashed onto ice. The solids were collected by Büchner filtration and dried under reduced pressure. The solid was then recrystallized from 85 mL of DCM and 30 mL of petroleum ether were added to induce crystallization. 12.38 g (31.0 mmol, 54 %) of 1,3,5-tris(bromomethyl)-2,4,6-trimethylbenzene (**2**) were obtained as white crystals.

^{1}H NMR (400 MHz, $CDCl_3$) δ [ppm]: 4.58 (s, 6H), 2.47 (s, 9H).

^{13}C NMR (100 MHz, CDCl3) δ [ppm]:138.06, 133.43, 30.05, 15.55.

HR-MS (DART) calculated for $[M+NH_4]^+$ 415.90517; found: 415.90401.

Spectroscopic data are in accordance with literature.[9]

2,4,6-Trimethyl-benzene-1,3,5-tricarbaldehyde

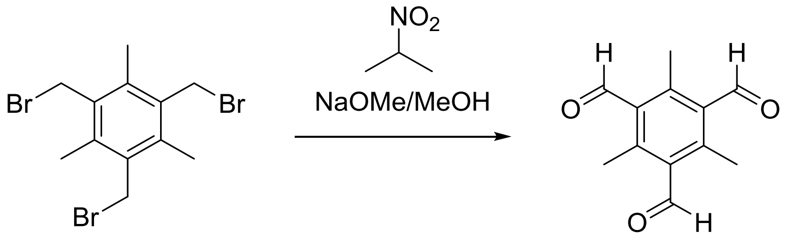

2 **3**

In a 500 mL round-bottom flask, 5.43 g (101 mmol, 4 equiv.) of sodium methoxide was dissolved in 290 mL of methanol. 13.3 mL (13.2 g, 148 mmol, 5.9 equiv.) of 2-nitropropane was added subsequently and the mixture was stirred for one hour at room temperature. Then, 10.00 g (25.07 mmol, 1 equiv.) of 1,3,5-tris(bromomethyl)-2,4,6-trimethylbenzene (**2**) was added and the mixture was stirred for another 22 h. After this time, 300 mL of water were added and the resulting white precipitate was isolated by Büchner filtration. The aqueous filtrate was extracted four times with 250 mL of dichloromethane and the organic layers were combined. The previous collected precipitate was dissolved in the organic layer

and the resulting solution was washed twice with 150 mL of water, before it was dried over anhydrous magnesium sulphate and filtered. The filtrate was concentrated to dryness under vacuum to afford the crude product as white solid. The crude product was further purified by filter column with dichloromethane (Rf = 0.33) to yield 3.91 g (19.16 mmol, 77 %) as a white solid.

^{1}H NMR (400 MHz, $CDCl_3$) δ [ppm]: 10.62 (s, 3H), 2.64 (s, 9H).

^{13}C NMR (100 MHz, CDCl3) δ [ppm]:194.60, 143.14, 134.97, 16.21.

HR-MS (ESI) calculated for $[M-H]^-$ 203.07137; found: 203.07064.

Spectroscopic data are in accordance with literature.[10]

COF synthesis:

The COF synthesis is based on a modified procedure of Smith *et al.*[11]:

General procedure:

The aldehyde (1 equiv.) and amine monomers (1.5 equiv.) were added to a 50 mL round bottom flask (RBF), together with a stirring rod, and were dissolved in a 4:1 *v/v* 1,4-dioxane:mesitylene mixture. The mixture was heated to 70 °C for 5 minutes to ensure dissolution. After cooling the mixture to approximately 40 °C, water and glacial acetic acid (for exact amounts, see the respective COF) were added. The reaction mixture was stirred at 70 °C for 3 days. Afterwards, the reaction was cooled to RT and the precipitate was collected via Büchner filtration. The solid was dispersed in dimethylformamide (DMF), stirred at 90 °C for 30 minutes and collected via Büchner filtration. These steps were repeated with DMF (90 °C, 30 min), ethanol (80 °C, 30 min), acetone (60 °C, 30 min) and hexane (70 °C, 30 min). After the final Büchner filtration, the COFs were divided over two petri-dishes and covered with tin foil for drying. The COFs were dried overnight at 120 °C, either in a regular oven or in a vacuum oven. After drying, the COFs were kept in the glovebox for storage.

Synthesis of TFB-PA COF

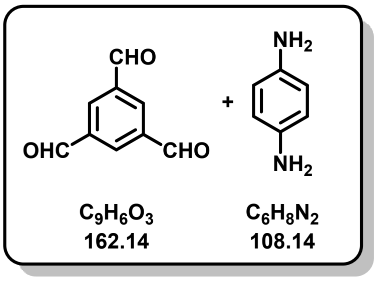

Table S1: The yield and the amount of monomers, solvent, water and acetic acid used for the synthesis of TFB PA at three scales, as well as the average BET surface areas of air- and vacuum-activated COFs.

	Scale 1	Scale 2	Scale 3
**Monomer mass aldehyde**	125 mg, 0.771 mmol (1 equiv.)	250 mg, 1.542 mmol (1 equiv.)	500 mg, 3.084 mmol (1 equiv.)
**Equivalent amine**	1.5	1.5	1.5
**1,4-Dioxane:mesitylene 4:1 *v/v* [mL]**	5	10	20
**H_2O**	1.2 mL 86.5 equiv.	2.4 mL 86.5 equiv.	4.8 mL 86.5 equiv.
**Glacial acetic acid**	1.8 mL (40.8 equiv.)	3.6 mL (40.8 equiv.)	7.2 mL (36.3 equiv.)
**Yield [%]**	68	81	/
**Average air-activated BET surface area [m²/g]**	1020±23		
**Average vacuum-activated BET surface area [m²/g]**	398±72		
**Average air stored BET surface are [m²/g]**	113±53		
**Scherrer analysis estimated domain size [nm]**	10.9±1.5		

Synthesis of TFB-BD COF

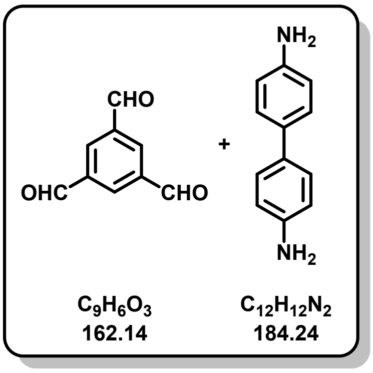

Table S2: The yield and the amount of monomers, solvent, water and acetic acid used for the syntheses of TFB BD, as well as the average BET surface areas of air and vacuum activated COFs.

	**Scale 1**	**Scale 2**
**Monomer mass aldehyde**	125 mg,<br>0.771 mmol<br>(1 equiv.)	250 mg,<br>1.542 mmol<br>(1 equiv.)
**Equivalent amine**	1.5	1.5
**1,4-Dioxane:mesitylene 4:1 *v/v* [mL]**	5	10
**H_2O**	1.2 mL<br>86.5 equiv.	2.4 mL<br>86.5 equiv.
**Glacial acetic acid**	1.8 mL<br>40.8 equiv.	3.6 mL<br>40.8 equiv.
**Yield [%]**	75	88
**Average air-activated BET surface area [m²/g]**	1514±97	
**Average vacuum-activated BET surface area [m²/g]**	1008±109	
**Average air stored BET surface are [m²/g]**	1530±33	
**Scherrer analysis estimated domain size [nm]**	15.1±0.4	

Synthesis of Me_3TFB-PA

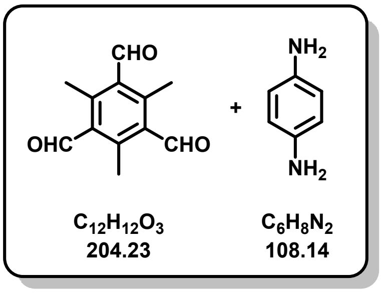

Table S3: The yield and the amount of monomers, solvent, water and acetic acid used for the syntheses of Me_3TFB PA, as well as the average BET surface areas of air and vacuum activated COFs.

	**Scale 1**	**Scale 2**
**Monomer mass aldehyde**	135 mg,<br>0.661 mmol<br>(1 equiv.)	250 mg,<br>1.224 mmol<br>(1 equiv.)
**Equivalent amine**	1.5	1.5
**1,4-Dioxane:mesitylene 4:1 *v/v* [mL]**	5	10
**H_2O**	1.2 mL<br>108.9 equiv.	2.4 mL<br>108.9 equiv.
**Glacial acetic acid**	1.8 mL<br>51.4 equiv.	3.0 mL<br>42.9 equiv.
**Yield [%]**	81	68
**Average air-activated BET surface area [m²/g]**	1877±20	
**Average vacuum-activated BET surface area [m²/g]**	2061±178	
**Average air stored BET surface are [m²/g]**	1973±158	
**Scherrer analysis estimated domain size [nm]**	15.1±1.5	

Synthesis of Me_3TFB-BD

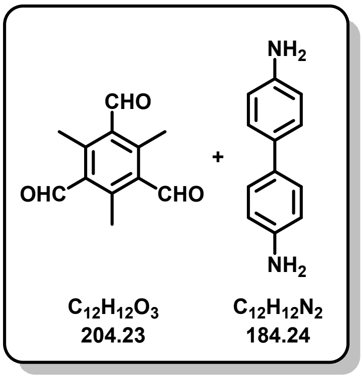

Table S4: The yield and the amount of monomers, solvent, water and acetic acid used for the syntheses of Me_3TFB BD, as well as the average BET surface areas of air and vacuum activated COFs.

	**Scale 1**	**Scale 2**
**Monomer mass aldehyde**	135 mg, 0.661 mmol (1 equiv.)	270 mg, 1.322 mmol (1 equiv.)
**Equivalent amine**	1.5	1.5
**1,4-Dioxane:mesitylene 4:1 *v/v* [mL]**	5	10
**H_2O**	1.2 mL 100.9 equiv.	2.4 mL 100.9 equiv.
**Glacial acetic acid**	1.5 mL 42.3 equiv.	3.2 mL 42.3 equiv.
**Yield [%]**	71	79
**Average air-activated BET surface area [m²/g]**	2115±50	
**Average vacuum-activated BET surface area [m²/g]**	2057±225	
**Average air stored BET surface are [m²/g]**	2291±219	
**Scherrer analysis estimated domain size [nm]**	20.5±2.1	

3. FT-IR Spectra

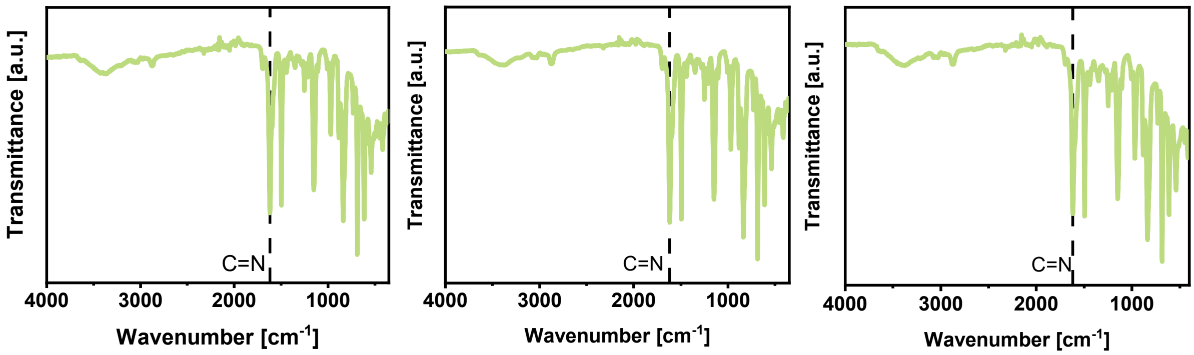

TFB-PA COF

Fig. S1: FT-IR spectra of all three synthesized TFB-PA COFs. The imine-stretch is at 1618 cm^{-1}.

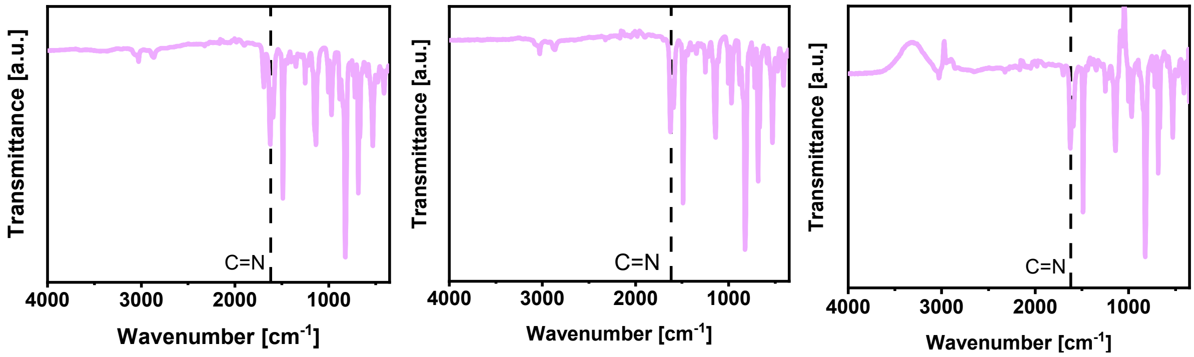

TFB-BD COF

Fig. S2: FT-IR spectra of all three TFB-BD COFs. The imine-stretch is at 1624 cm^{-1}.

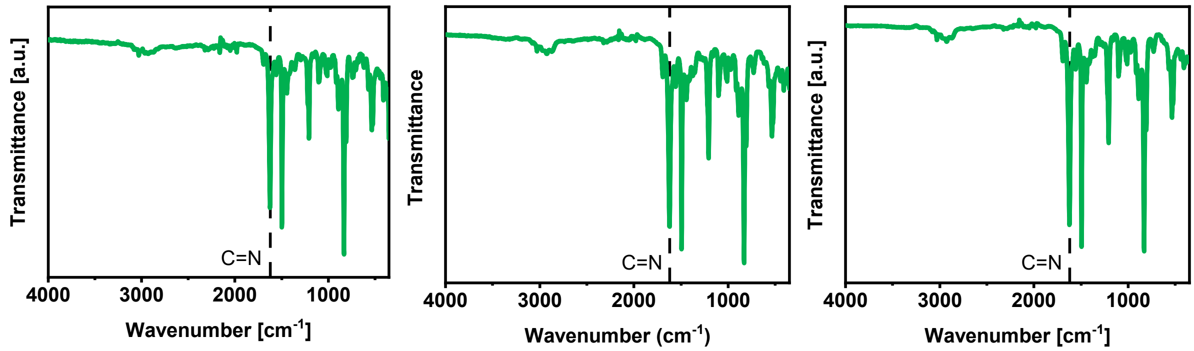

Me_3TFB-PA COF

Fig. S3: FT-IR spectra of all three synthesized Me_3TFB-PA COFs. The imine-stretch is at 1623 cm^{-1}.

Me_3TFB-BD COF

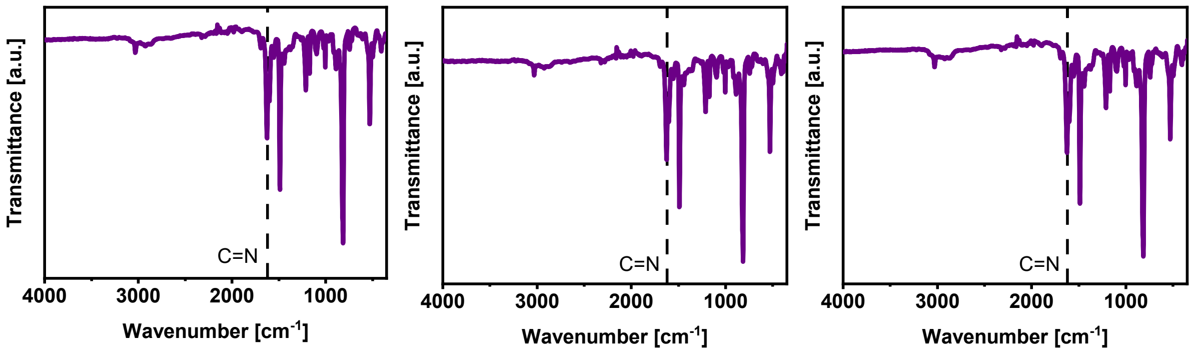

Fig. S4: FT-IR spectra of all three synthesized Me_3TFB-BD COFs. The imine-stretch is at 1627 cm^{-1}.

4. Powder X-Ray Diffraction Analysis

All FT-IR spectra indicate that the synthesis was repeatable which is also confirmed by nitrogen sorption measurements. Therefore, only one representative PXRD and ssNMR spectrum of the triplicates will be displayed.

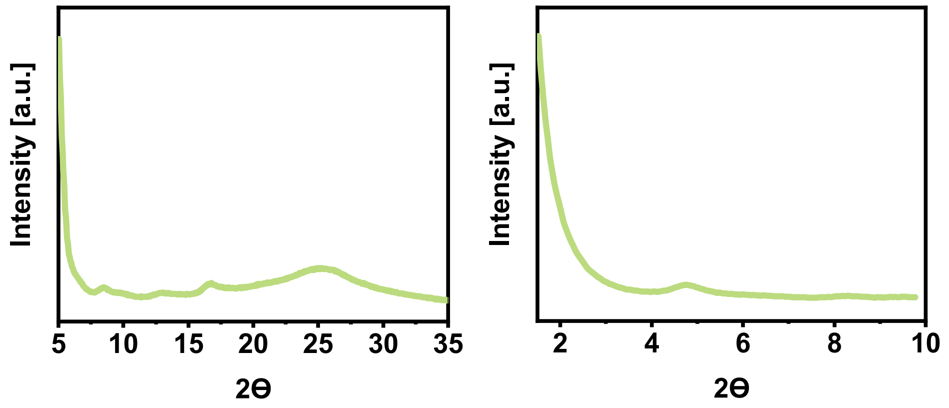

TFB-PA COF

Fig. S5: PXRD spectrum between 4-35° (left) of TFB-BD COF. The diffraction pattern matches with literature.[12]

TFB-BD COF

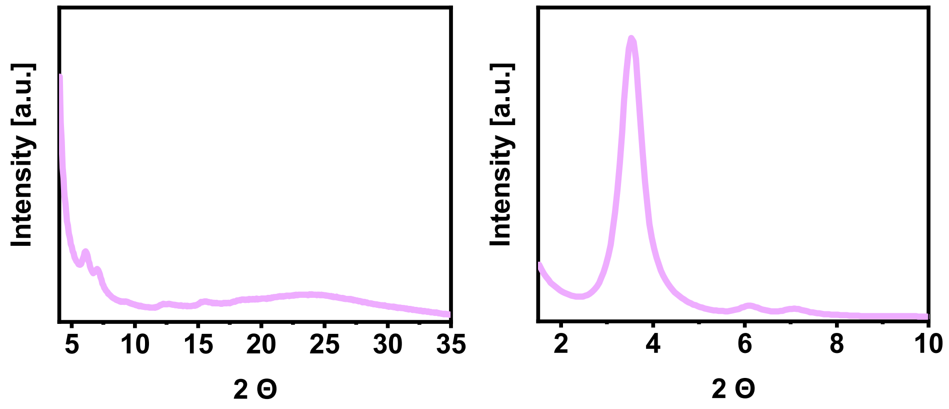

Fig. S6: PXRD spectrum between 4-35° (left) and 1-10°(right) of TFB-BD COF. The diffraction pattern matches with literature.[13,14]

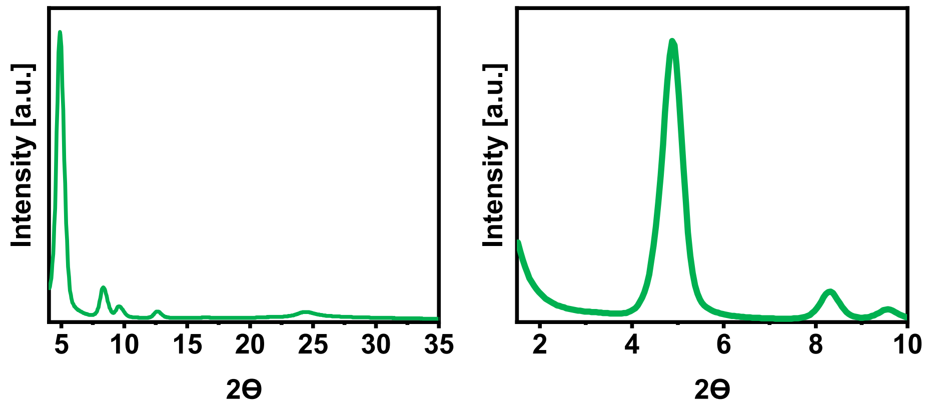

Me_3TFB-PA COF

Fig. S7: PXRD spectrum between 4-35° (left) and 1-10°(right) of Me_3TFB-PA COF.

Me_3TFB-BD COF

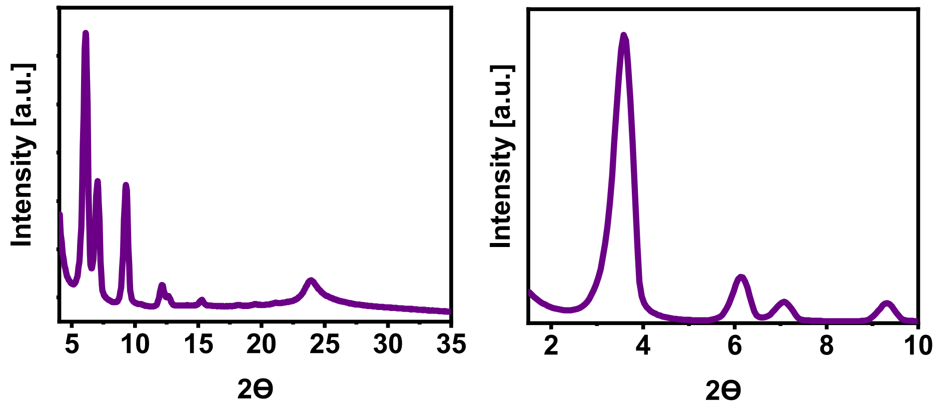

Fig. S8: PXRD spectrum between 4-35° (left) and 1-10°(right) of Me_3TFB-BD COF.

5. ^{13}C CPMAS NMR Spectra

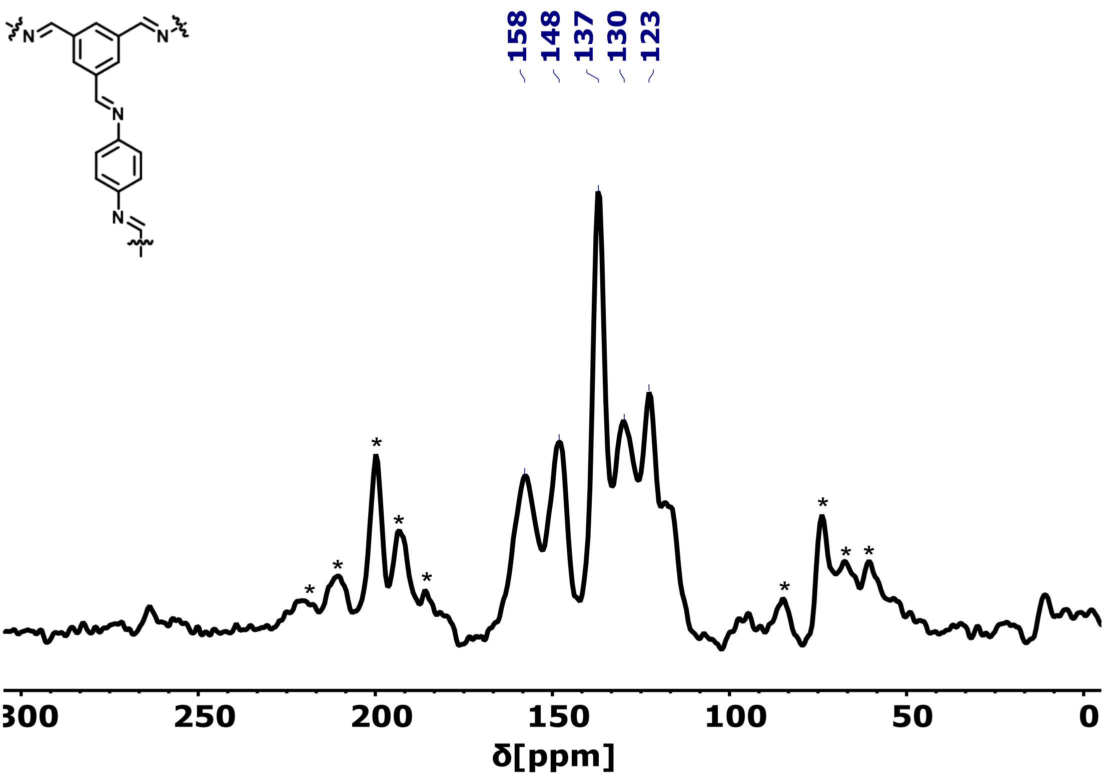

Fig. S9: Solid-state NMR spectrum of TFB-PA COF. The signal at 158 ppm indicates the formation of imine bonds. Spinning side bands were determined by comparing different MAS frequencies.

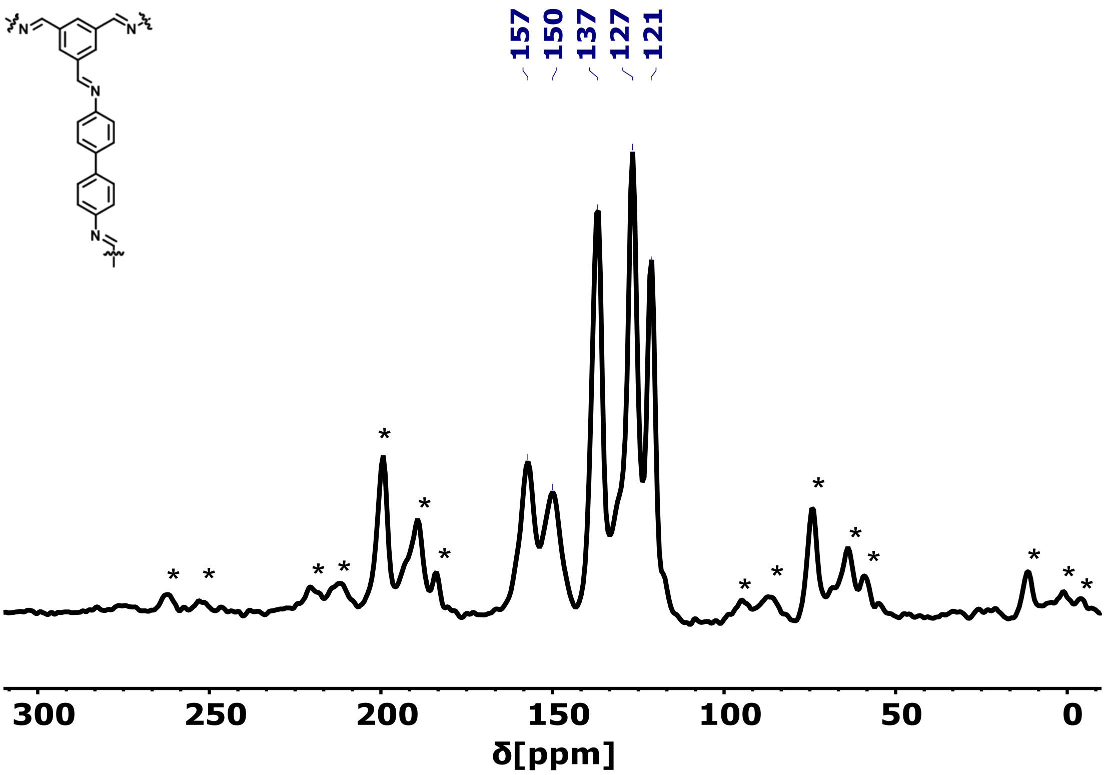

Fig. S10: Solid-state NMR spectrum of TFB-BD COF. The signal at 157 ppm indicates the formation of imine bonds. Spinning side bands were determined by comparing different MAS frequencies.

6. Nitrogen Sorption Analysis

All COFs have been synthesized three times and were then divided in two batches for different activation methods:

1. 120 °C, oven-dried, overnight
2. 120 °C, vacuum-oven-dried, overnight

For all samples nitrogen sorption analysis was carried out and the BET surface area has been calculated. The range for the linear regression has been the same for all repetitions of the same COF and for both activation methods and to determine the surface area for the air stability after four weeks. The same DFT model has been used for one COF structure to determine the pore size distribution. All graphs displayed are representative for the respective COF.

TFB-PA COF

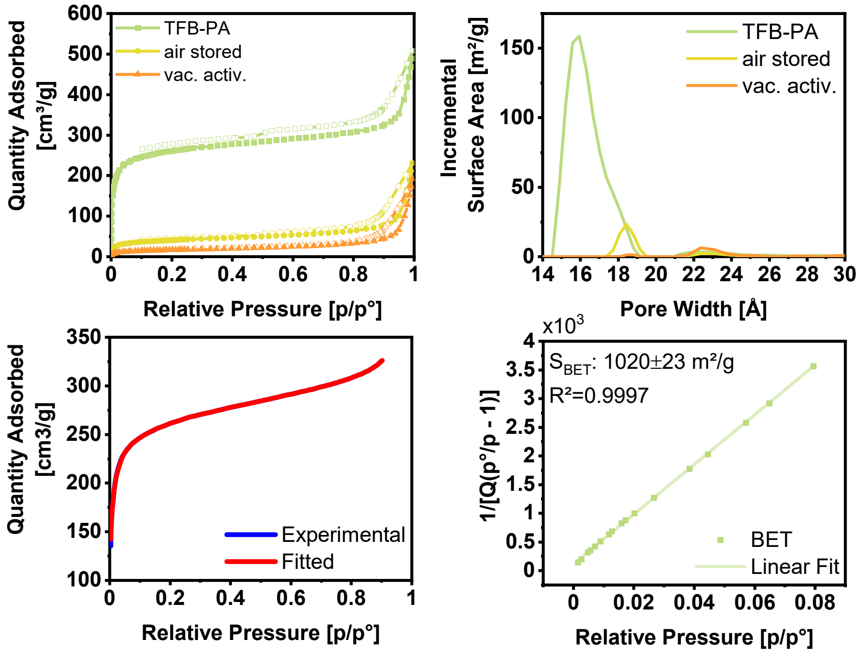

Fig. S11: Top left: representative adsorption (solid symbols)-desorption (open symbols) isotherms of TFB-PA COF for oven-dried samples (light green), vacuum-dried samples (orange) and stored for 4 weeks on the bench (yellow), top right: pore size distribution of TFB-PA COF oven-dried (light green), vacuum-dried (orange) and after 4 weeks on the bench (yellow), bottom left: comparison of the experimental adsorption isotherm with the theoretically modelled isotherm, bottom right: linear fit to calculate the BET surface area, including R^2.

TFB-BD COF

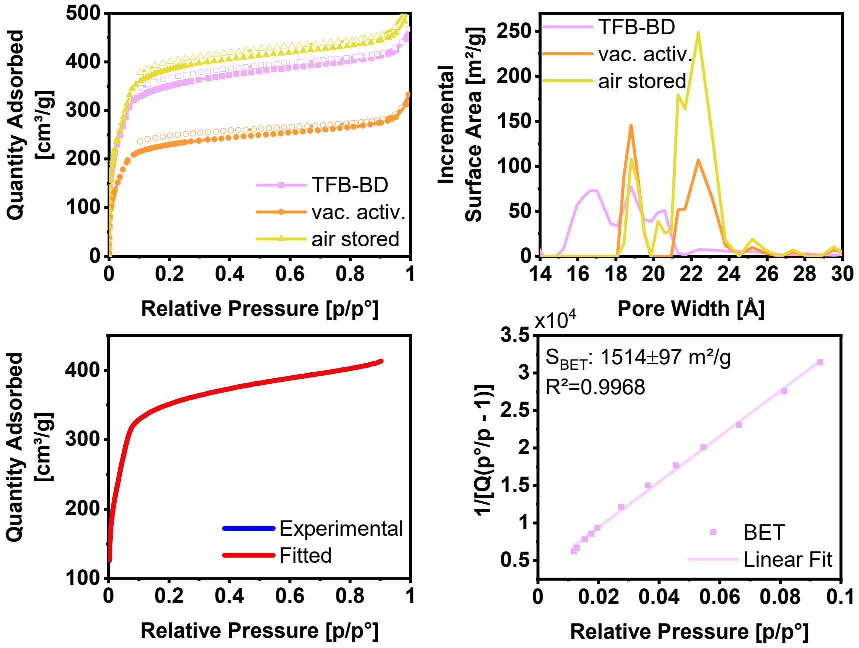

Fig. S12: Top left: representative adsorption (solid symbols)-desorption (open symbols) isotherms of TFB-BD COF for oven-dried samples (pink), vacuum-dried samples (orange) and stored for 4 weeks on the bench (yellow), top right: pore size distribution of TFB-BD COF oven-dried (pink), vacuum-dried (orange) and after 4 weeks on the bench (yellow), bottom left: comparison of the experimental adsorption isotherm with the theoretically modelled isotherm, bottom right: linear fit to calculate the BET surface area, including R^2.

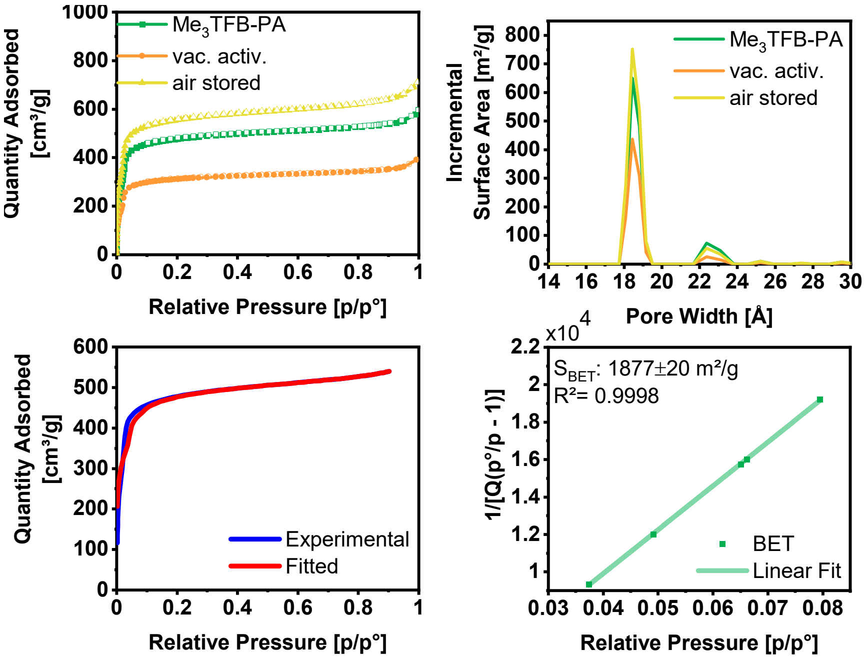

Me_3TFB-PA COF

Fig. S13: Top left: representative adsorption (solid symbols)-desorption (open symbols) isotherms of Me_3TFB-PA COF for oven-dried samples (dark green), vacuum-dried samples (orange) and stored for 4 weeks on the bench (yellow), top right: pore size distribution of Me_3TFB-PA COF oven-dried (dark green), vacuum-dried (orange) and after 4 weeks on the bench (yellow), bottom left: comparison of the experimental adsorption isotherm with the theoretically modelled isotherm, bottom right: linear fit to calculate the BET surface area, including R^2.

Me_3TFB-BD COF

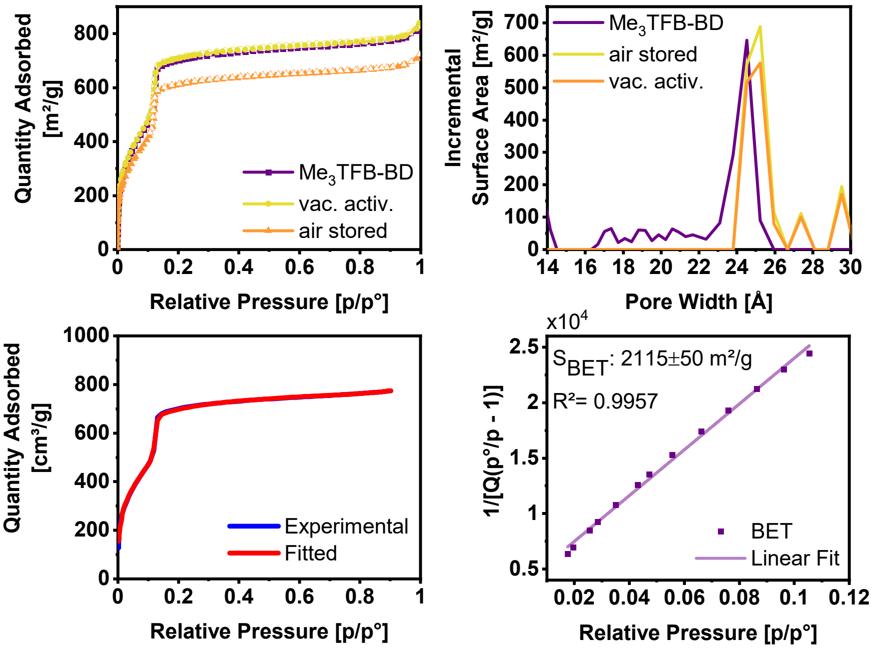

Fig. S14: Top left: representative adsorption (solid symbols)-desorption (open symbols) isotherms of Me_3TFB-BD COF for oven-dried samples (purple), vacuum-dried samples (orange) and stored for 4 weeks on the bench (yellow), top right: pore size distribution of Me_3TFB-BD COF oven-dried (purple), vacuum-dried (orange) and after 4 weeks on the bench (yellow), bottom left: comparison of the experimental adsorption isotherm with the theoretically modelled isotherm, bottom right: linear fit to calculate the BET surface area, including R^2.

7. Thermogravimetric analysis

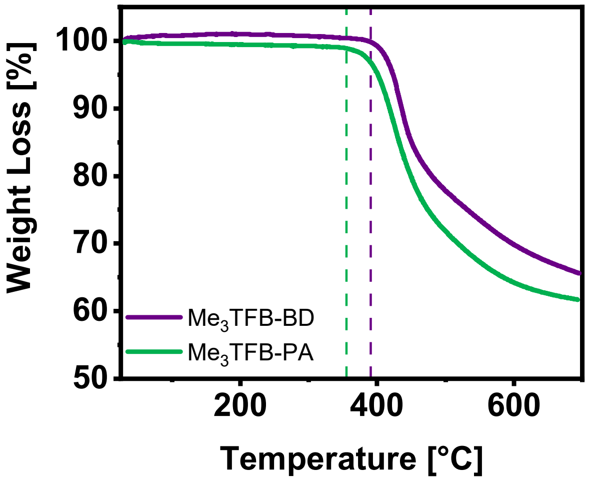

Fig. S15: Thermogravimetric analysis of Me_3TFB-PA and Me_3TFB-BD COF. The COFs are stable up to 355 °C and 390 °C, respectively.

8. DFT modelling

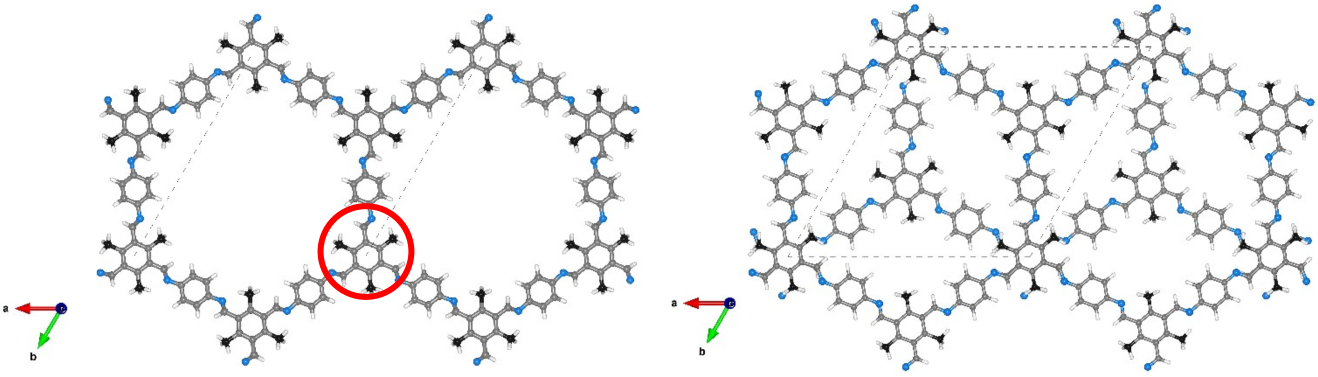

Fig. S16: DFT-optimized crystal structures of Me_3TFB-PA: eclipsed conformation (left) and staggered conformation (right). The aldehyde node is C_3 symmetric within the framework (red circle). Coordinates available as separate files.

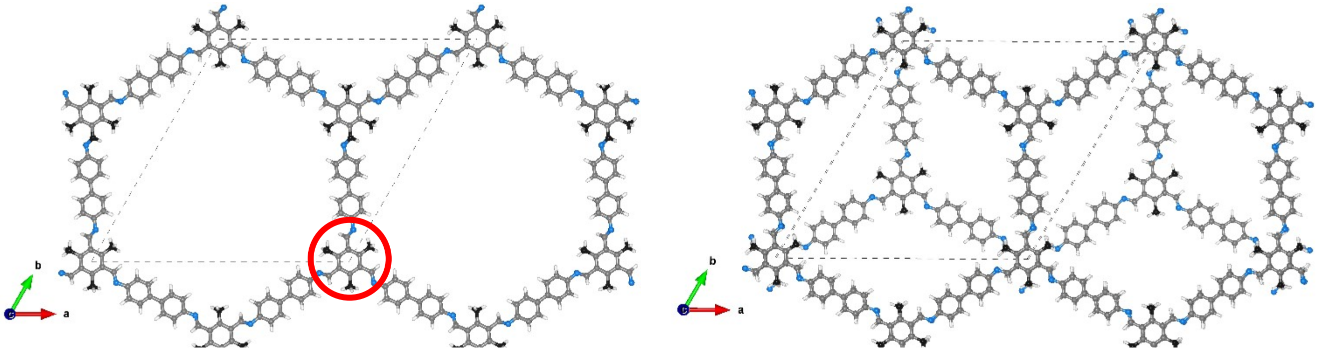

Fig. S17: DFT-optimized crystal structures of Me_3TFB-BD: eclipsed conformation (left) and staggered conformation (right). The aldehyde node is C_3 symmetric within the framework (red circle). Coordinates available as separate files.

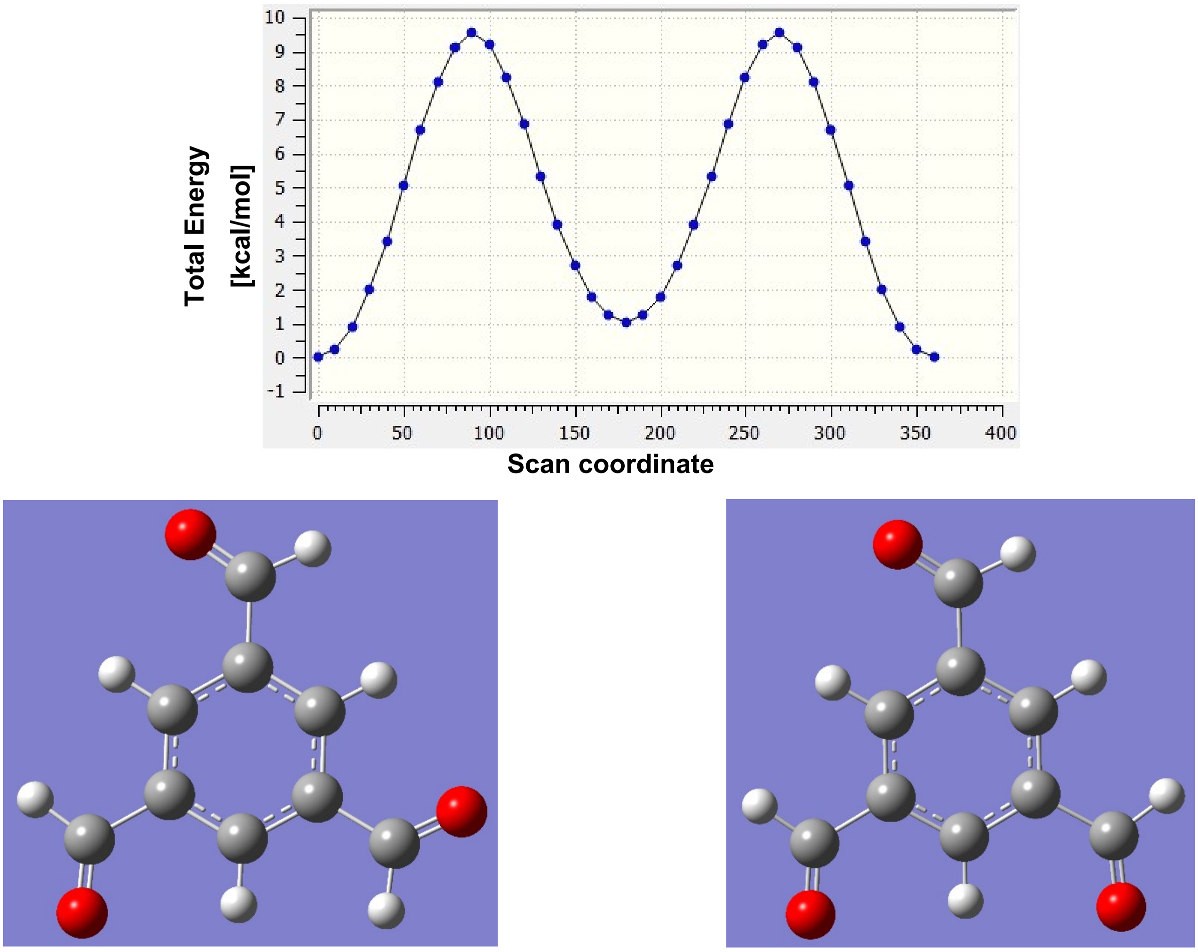

Fig. S18: Conformational energy diagram of the carbonyl group of TFB as a function of the dihedral angle (top) with their respective structures of the minima (bottom). The absolute minimum is the left structure and the local minimum the right structure. The absolute energy of the left structure was set to 0 kcal/mol.

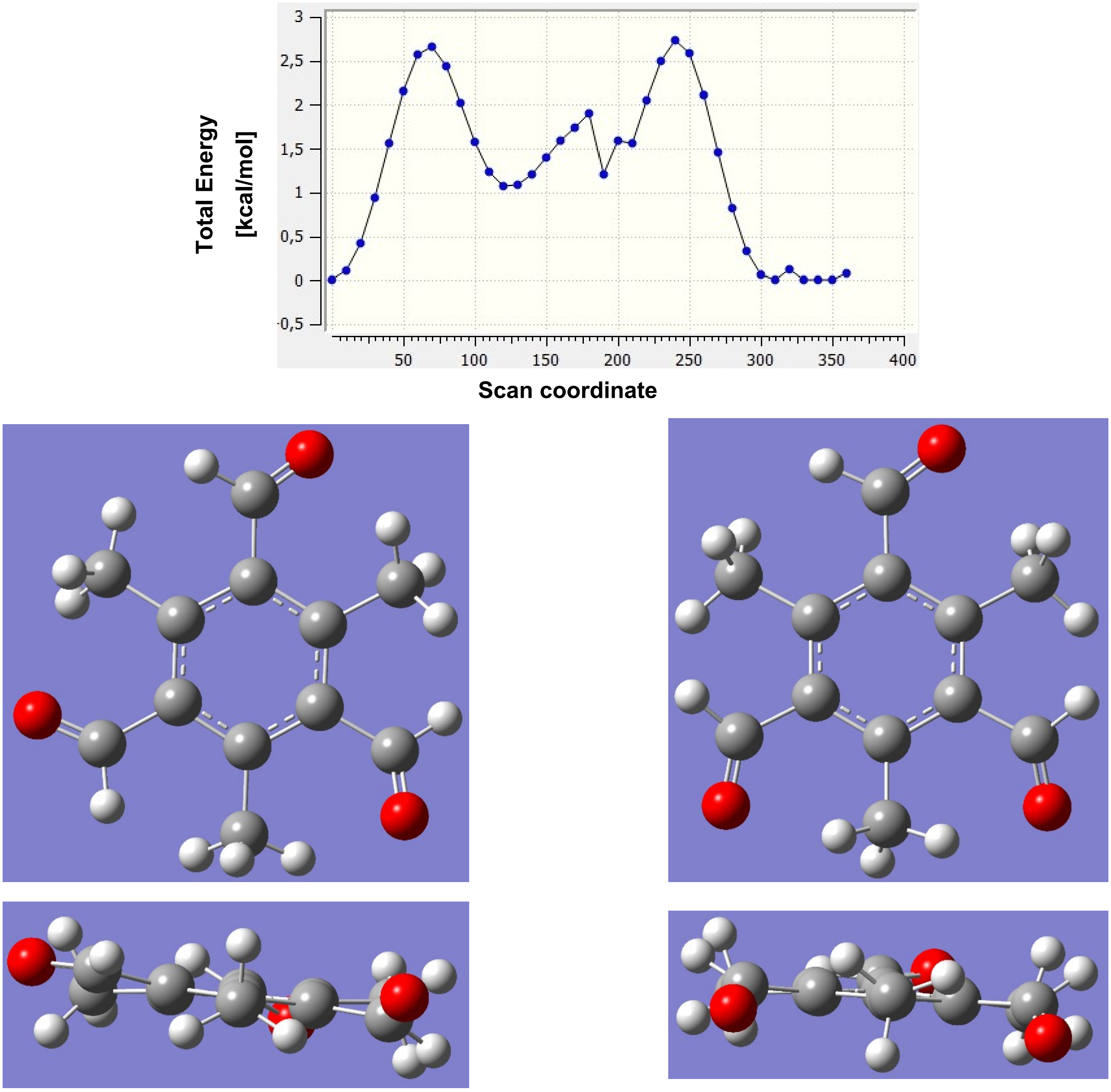

Fig. S19: Conformational energy diagram of the carbonyl group of Me_3TFB as a function of the dihedral angle (top) with their respective structures of the minima (middle, bottom). The absolute minimum is the left structure and the local minimum the right structure. The aromatic ring and the carbonyl groups are not perfectly conjugated. The absolute energy of the left structure was set to 0 kcal/mol.

For the rate constant of the conformation equilibrium, we took the Arrhenius equation (Equation 1) and assumed the same pre-exponential factor *A*, because TFB and Me_3TFB are similar in their structure.

$$k = A \times e^{-\frac{E_a}{RT}} \tag{1}$$

Using 70 °C (343.15 K) as the reaction temperature, R = 8.314 J K^{-1} mol^{-1}, and ΔE_a = 29820 J mol^{-1} the difference in rate constants is:

$\Delta k = 3 \times 10^5\ s^{-1}$

Calculation of the formation energy:

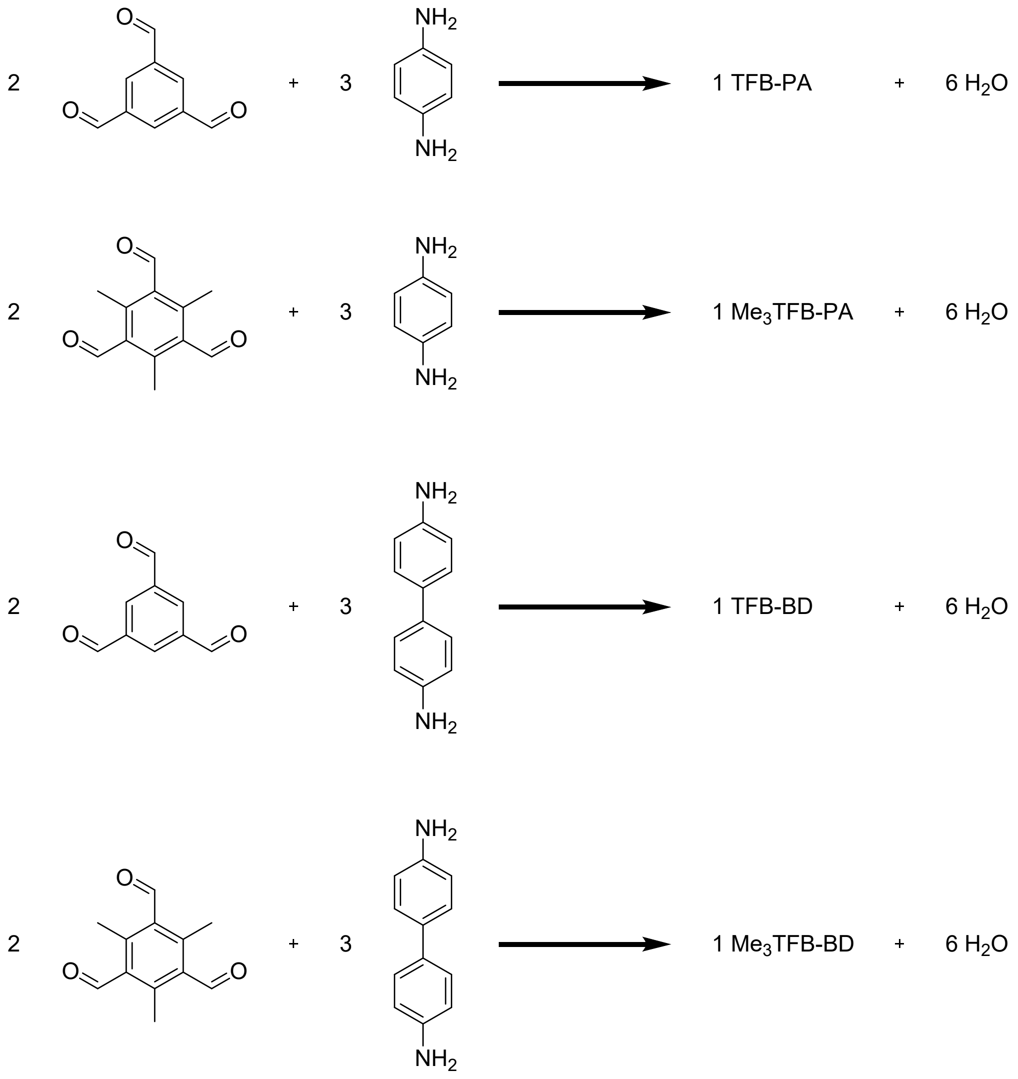

Fig. S20: Balanced reaction schemes to determine the formation energy for each COF.

Table S5: DFT-computed formation energies of all COFs.

COF	Energy [eV]	Energy [kcal/mol]
**TFB-PA**	-3.87	-89.2443
**Me_3TFB-PA**	-5.40	-124.527
**TFB-BD**	-5.28	-121.76
**Me_3TFB-BD**	-6.35	-146.434

9. Vapor Experiments

COF powder is added into an empty tea bag and the tea bag is closed with tape. The tea bag is added to a flask and hydrochloric acid enriched vapor (argon bubbled through a flask filled with concentrated hydrochloric acid heated to 50 °C) is flushed through the flask for 30 min. Afterwards, the vapor stream is removed, the COF powder taken out of the tea bag and the absorption spectrum is measured.

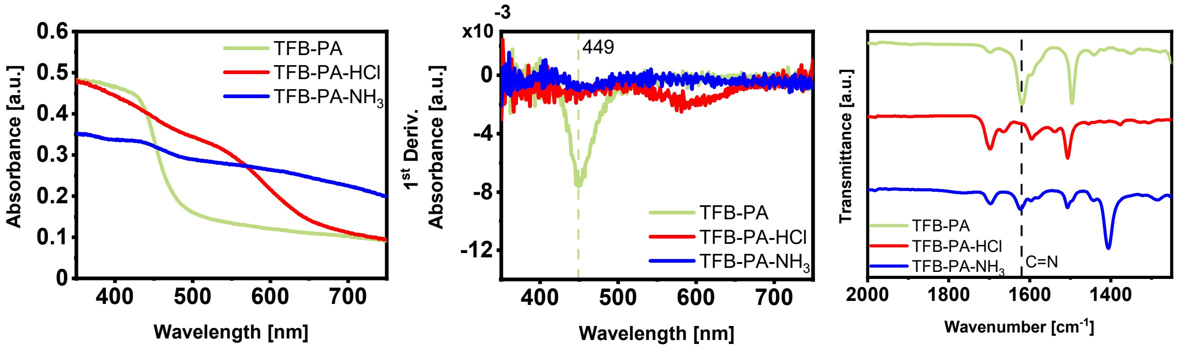

Fig. S21: UV/vis absorbance spectra (left) of TFB-PA before (light green) and after hydrochloric acid exposure (red) and after subsequent ammonia exposure (blue). The first derivatives are shown on the right and the FT-IR of each sample in the bottom.

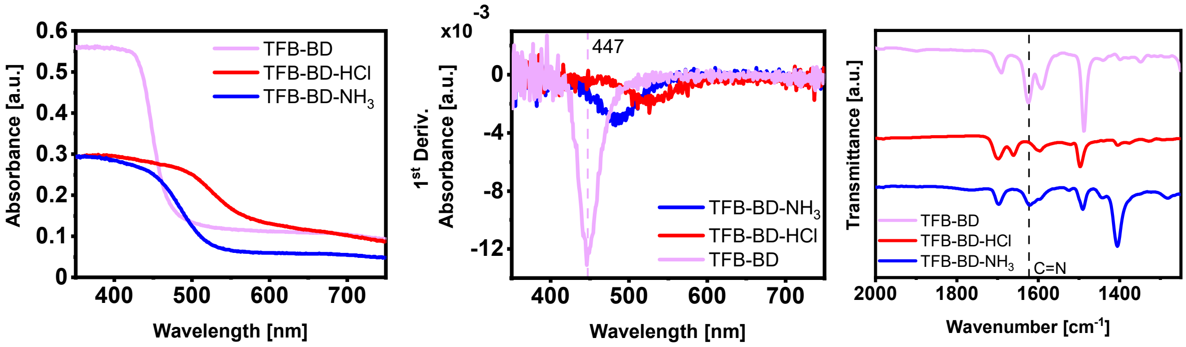

Fig. S22: UV/vis absorbance spectra (left) of TFB-BD before (light purple) and after hydrochloric acid exposure (red) and after subsequent ammonia exposure (blue). The first derivatives are shown on the right and the FT-IR of each sample in the bottom.

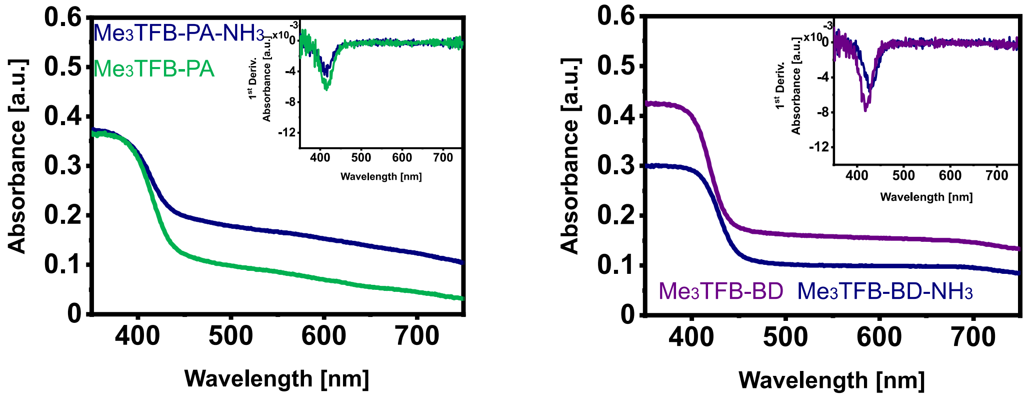

Fig. S23: UV/Vis absorbance of Me_3TFB-PA (left) and Me_3TFB-BD (right) in comparison to ammonia-exposed COF samples. The inset is the first derivative.

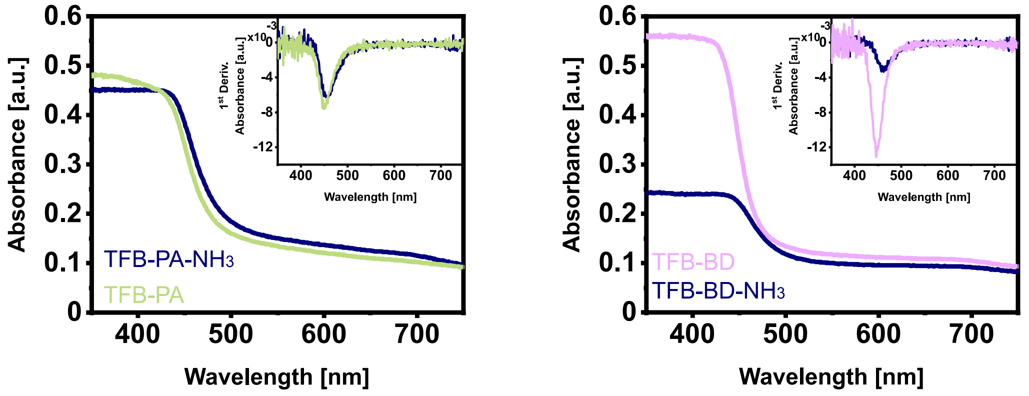

Fig. S24: UV/Vis absorbance of TFB-PA (left) and TFB-BD (right) in comparison to ammonia-exposed COF samples. The inset is the first derivative.

10. ^{1}H-NMR and ^{13}C-NMR spectra of building blocks

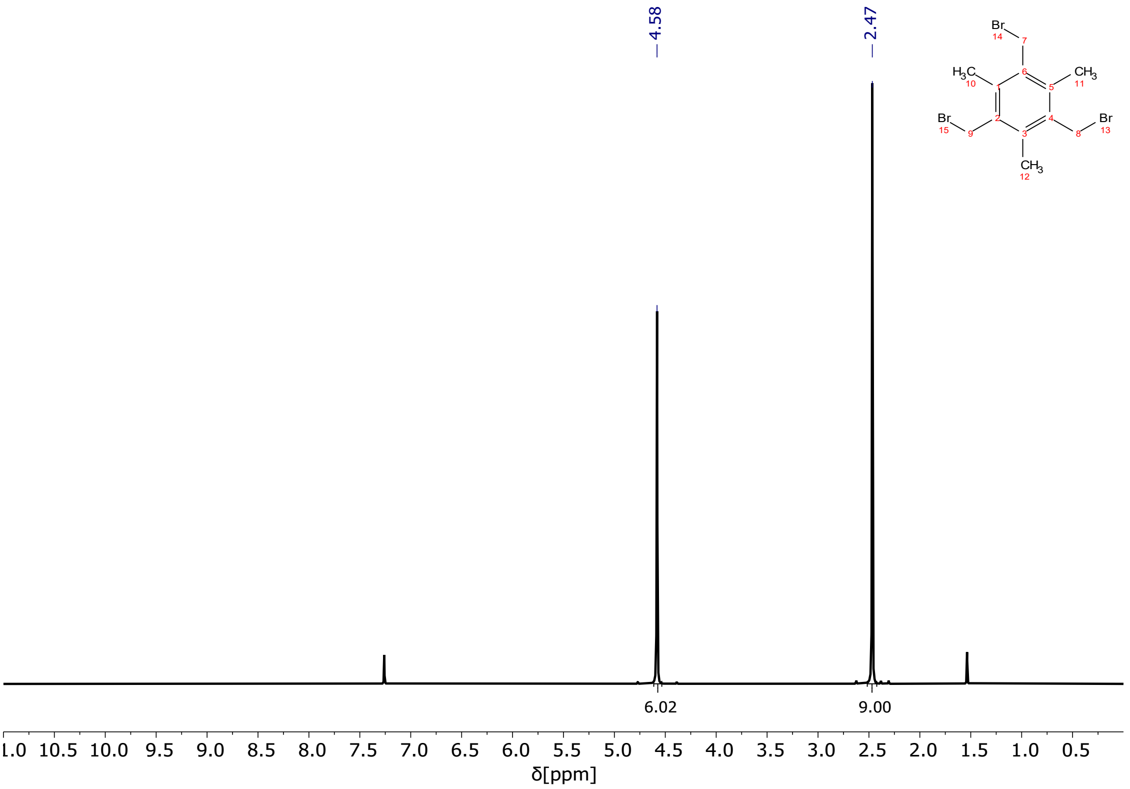

Fig. S25: ^{1}H-NMR spectrum of 1,3,5-tris(bromomethyl)-2,4,6-trimethylbenzene in $CDCl_3$ at 400 MHz.

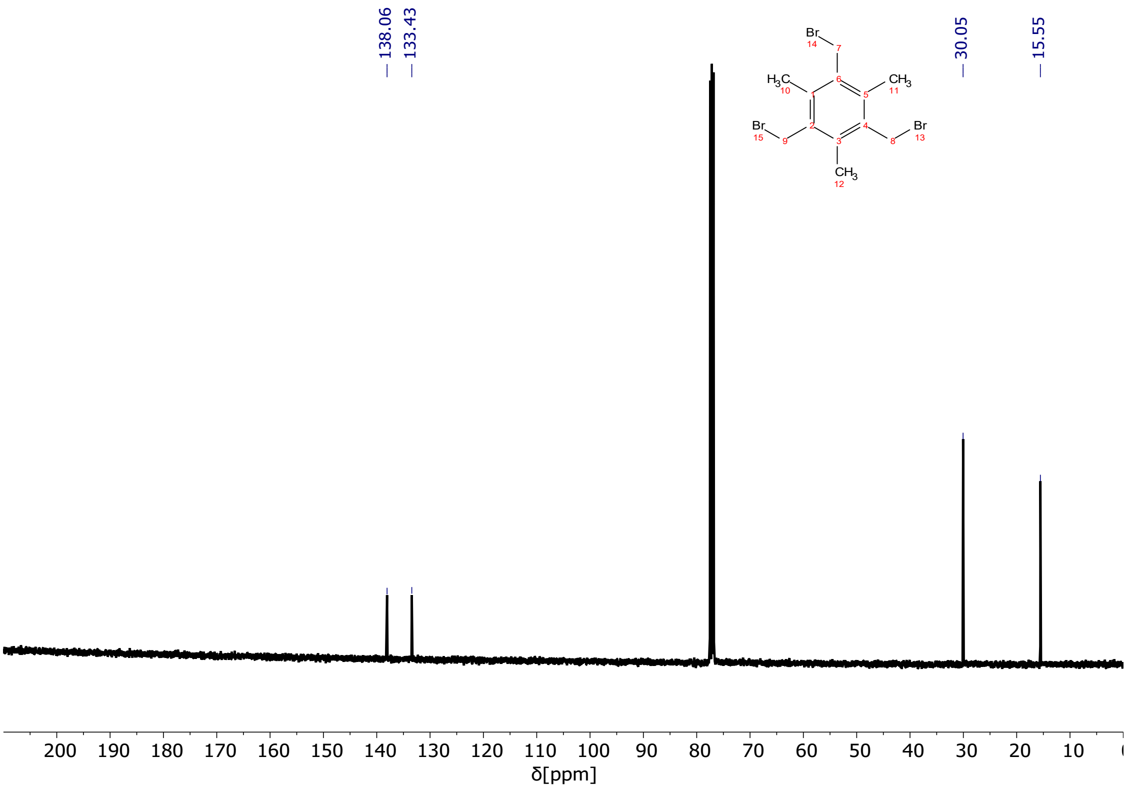

Fig. S26: ^{13}C-NMR spectrum of 1,3,5-tris(bromomethyl)-2,4,6-trimethylbenzene in $CDCl_3$ at 100 MHz.

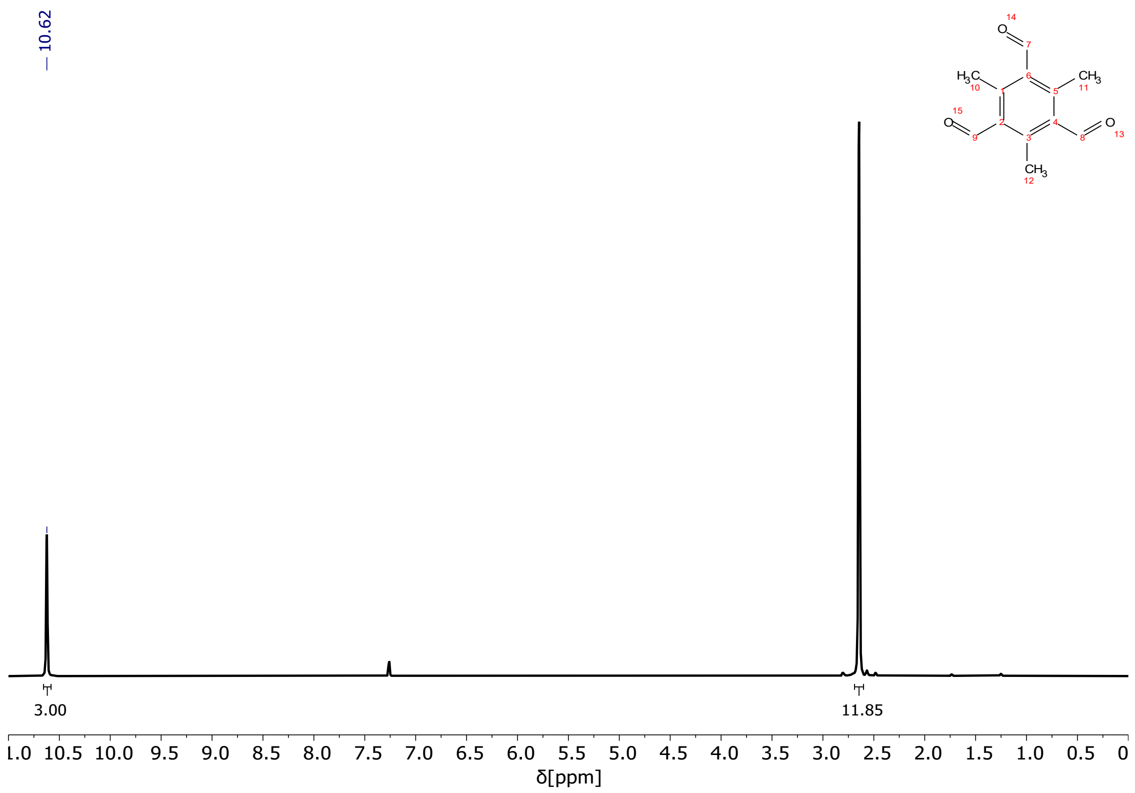

Fig. S27: ^{1}H-NMR spectrum of 2,4,6-trimethyl-benzene-1,3,5-tricarbaldehyde in $CDCl_3$ at 400 MHz.

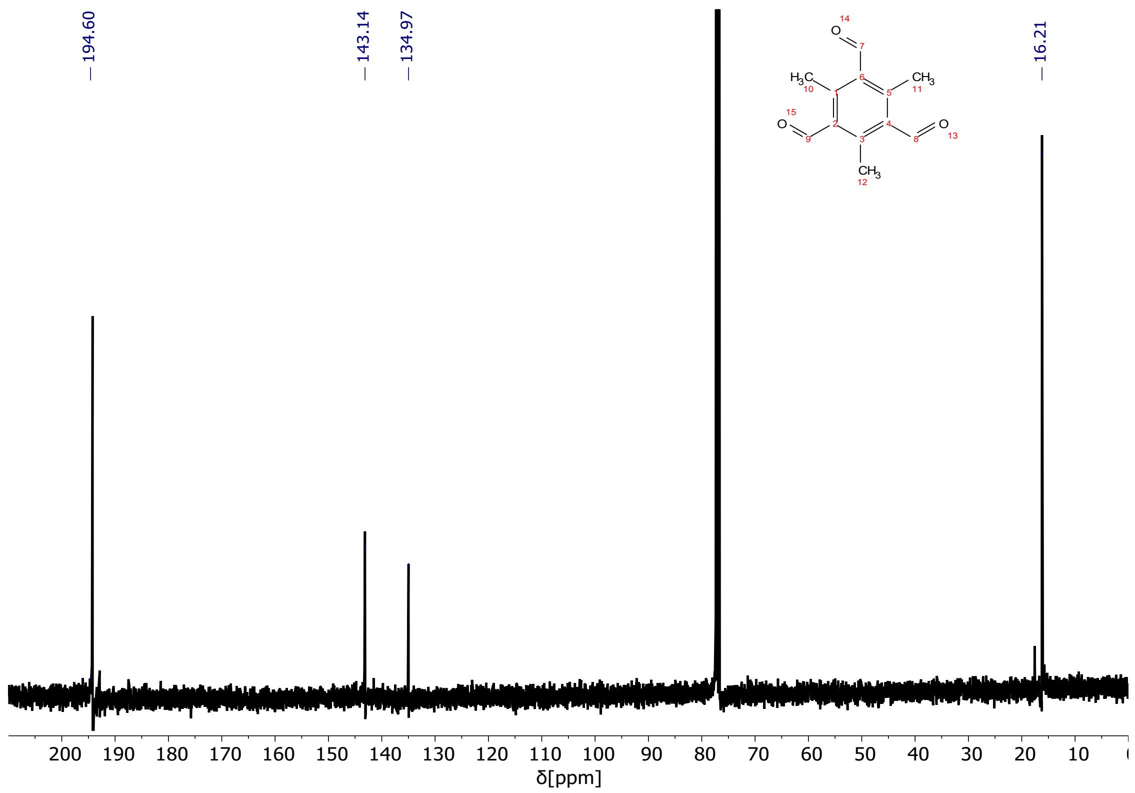

Fig. S28: ^{13}C-NMR spectrum of 2,4,6-trimethyl-benzene-1,3,5-tricarbaldehyde in $CDCl_3$ at 100 MHz.

11. Mass Spectrometry

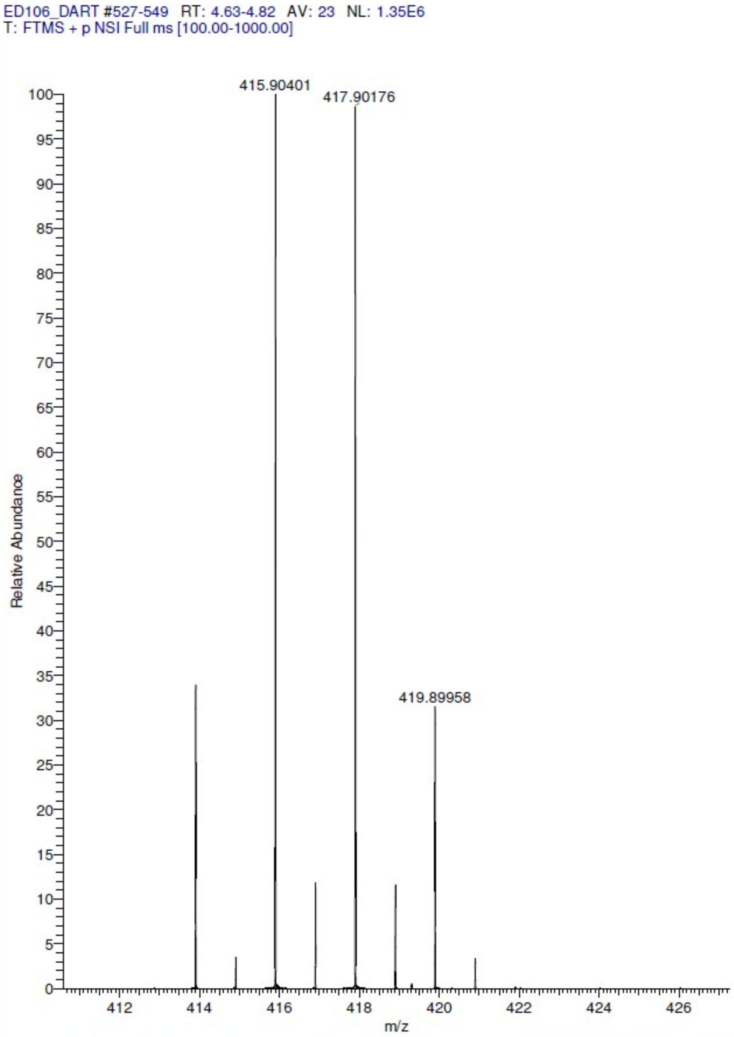

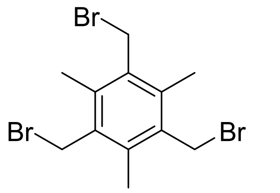

calculated for $[M+NH_4]^+$ 415.90517

Fig. S29: HR-MS spectrum of 1,3,5-tris(bromomethyl)-2,4,6-trimethylbenzene.

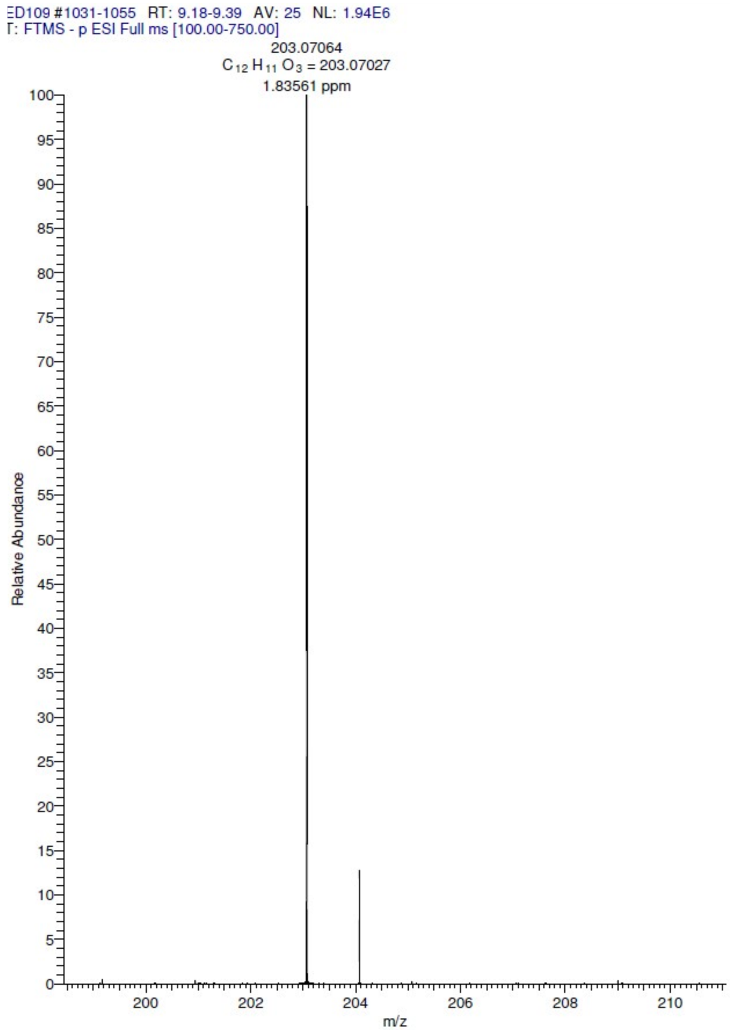

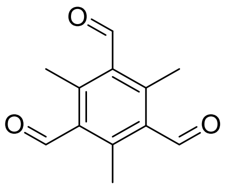

calculated for [M-H]- 203.07137

Fig. S30: HR-MS spectrum for 2,4,6-trimethyl-benzene-1,3,5-tricarbaldehyde.

12. References

1 M. Loos, C. Gerber, F. Corona, J. Hollender and H. Singer, *Anal. Chem.*, 2015, **87**, 5738–5744.

2 G. Kresse and J. Furthmüller, *Phys. Rev. B - Condens. Matter Mater. Phys.*, 1996, **54**, 11169–11186.

3 D. Kresse G, Joubert, *Phys. Rev. B - Condens. Matter Mater. Phys.*, 1999, **59**, 1758–1775.

4 J. P. Perdew, K. Burke and M. Ernzerhof, *Phys. Rev. Lett.*, 1996, **77**, 3865–3868.

5 P. E. Blöchl, *Phys. Rev. B*, 1994, **50**, 17953–17979.

6 S. Grimme, S. Ehrlich and L. Goerigk, *J. Comput. Chem.*, 2011, **32**, 1456–1465.

7 K. Momma and F. Izumi, *J. Appl. Crystallogr.*, 2011, **44**, 1272–1276.

8 Gaussian 16, Revision C.01, M. J. Frisch, G. W. Trucks, H. B. Schlegel, G. E. Scuseria, M. A. Robb, J. R. Cheeseman, G. Scalmani, V. Barone, G. A. Petersson, H. Nakatsuji, X. Li, M. Caricato, A. V Marenich, J. Bloino, B. G. Janesko, R. Gomperts, B. Mennucci, H. P. Hratchian, J. V Ortiz, A. F. Izmaylov, J. L. Sonnenberg, D. Williams-Young, F. Ding, F. Lipparini, F. Egidi, J. Goings, B. Peng, A. Petrone, T. Henderson, D. Ranasinghe, V. G. Zakrzewski, J. Gao, N. Rega, G. Zheng, W. Liang, M. Hada, M. Ehara, K. Toyota, R. Fukuda, J. Hasegawa, M. Ishida, T. Nakajima, Y. Honda, O. Kitao, H. Nakai, T. Vreven, K. Throssell, J. A. Montgomery Jr., J. E. Peralta, F. Ogliaro, M. J. Bearpark, J. J. Heyd, E. N. Brothers, K. N. Kudin, V. N. Staroverov, T. A. Keith, R. Kobayashi, J. Normand, K. Raghavachari, A. P. Rendell, J. C. Burant, S. S. Iyengar, J. Tomasi, M. Cossi, J. M. Millam, M. Klene, C. Adamo, R. Cammi, J. W. Ochterski, R. L. Martin, K. Morokuma, O. Farkas, J. B. Foresman and D. J. Fox, Gaussian Inc. Wallingford CT, 2016.

9 A. W. van der Made and R. H. van der Made, *J. Org. Chem.*, 1993, 58, 1262–1263.

10 A. G. Slater, P. S. Reiss, A. Pulido, M. A. Little, D. L. Holden, L. Chen, S. Y. Chong, B. M. Alston, R. Clowes, M. Haranczyk, M. E. Briggs, T. Hasell, G. M. Day and A. I. Cooper, *ACS Cent. Sci.*, 2017, **3**, 734–742.

11 B. J. Smith, A. C. Overholts, N. Hwang and W. R. Dichtel, *Chem. Commun.*, 2016, **52**, 3690–3693.

12 S.-Y. Ding, J. Gao, Q. Wang, Y. Zhang, W.-G. Song, C.-Y. Su and W. Wang, *J. Am. Chem. Soc.*, 2011, **133**, 19816–19822.

13 L. Bai, S. Z. F. Phua, W. Q. Lim, A. Jana, Z. Luo, H. P. Tham, L. Zhao, Q. Gao and Y. Zhao, *Chem. Commun.*, 2016, **52**, 4128–4131.

14 Y. Yang, D. Deng, S. Zhang, Q. Meng, Z. Li, Z. Wang, H. Sha, R. Faller, Z. Bian, X. Zou, G. Zhu and Y. Yuan, *Adv. Mater.*, 2020, **32**, 1908243.
